# An Investigation on Attributes of Ambient Temperature and Diurnal Temperature Range on Mortality in Five East-Asian Countries

**DOI:** 10.1038/s41598-017-10433-8

**Published:** 2017-08-31

**Authors:** Whan-Hee Lee, Youn-Hee Lim, Tran Ngoc Dang, Xerxes Seposo, Yasushi Honda, Yue-Liang Leon Guo, Hye-Min Jang, Ho Kim

**Affiliations:** 1 Graduated School of Public Health, Seoul National University, Seoul, Republic of Korea; 2 Institute of Environmental Medicine, Seoul National University of Medical Research Center, Seoul National University, Seoul, Republic of Korea; 3 Environmental Health Center, Seoul National University College of Medicine, Seoul, Republic of Korea; 4 Faculty of Health and Sports Sciences, University of Tsukuba, Tsukuba, Japan; 5 Department of Environmental and Occupational Medicine, National Taiwan University, Taipei, Taiwan; 6 Department of Statistics, Ewha Womans’ University, Seoul, Republic of Korea

## Abstract

Interest in the health effects of extremely low/high ambient temperature and the diurnal temperature range (DTR) on mortality as representative indices of temperature variability is growing. Although numerous studies have reported on these indices independently, few studies have provided the attributes of ambient temperature and DTR related to mortality, concurrently. In this study, we aimed to investigate and compare the mortality risk attributable to ambient temperature and DTR. The study included data of 63 cities in five East-Asian countries/regions during various periods between 1972 and 2013. The attributable risk of non-accidental death to ambient temperature was 9.36% (95% confidence interval [CI]: 8.98–9.69%) and to DTR was 0.59% (95% CI: 0.53–0.65%). The attributable cardiovascular mortality risks to ambient temperature (15.63%) and DTR (0.75%) are higher than the risks to non-accidental/respiratory-related mortality. We verified that ambient temperature plays a larger role in temperature-associated mortality, and cardiovascular mortality is susceptible to ambient temperature and DTR.

## Introduction

In general, ambient temperature and sudden temperature change have been reported as the prominent causes of weather-related mortality^1–4^. Moreover, the Intergovernmental Panel on Climate Change (IPCC) recently reported greenhouse gases will evidently increase the earth’s ambient temperature and predicted climate change would cause frequent weather pattern instability (e.g., rapid increase/decrease of temperature)^5^. As a result, the importance of assessing the effect of ambient temperature and temperature change on health is heightened.

Some researchers have found that the exposure–response relationship of temperature presents a U- or V-shaped association^1, 3^, and previous studies have found epidemiological evidence that the risk of mortality increases when the temperature is extremely high or low^6, 7^. In addition, many studies also reported the diurnal temperature range (DTR) (e.g., intra-day temperature change is an index representing sudden temperature change within a day, which is calculated by subtracting the minimum temperature from the maximum temperature) as one of the environmental risk factors of mortality (i.e., non-accidental, cardiovascular-related, and respiratory-related deaths) or morbidity^8–11^ in Asia^12, 13^ and North America^2, 14^.

Although concerns about climate change have been numerous, including the increases in DTR, many epidemiological studies have separately reported on the health impact of the low/high temperature and DTR^1, 15–17^. Even if these three measures (low temperature, high temperature, and DTR) are widely used indices of temperature variability, the complex correlational effects of temperature and its variability on mortality are still undetermined^17^. Since people are exposed to ambient temperature and DTR simultaneously, studies also need to consider these effects concurrently.

In this study, we aimed to comprehensively investigate and compare the attributable risks of temperature and DTR on three specific causes of mortality, non-accidental, cardiovascular-related, and respiratory-related, among Asian countries/regions. We considered attributable risk fractions of temperature indices using an advanced statistical method, the distributed lag non-linear model (DLNM)^18^, and applied it to 63 cities in five East Asian countries/regions (Japan, South Korea [hereafter Korea], Chinese Taiwan, Vietnam, and the Philippines), which are exposed to different weather conditions.

## Results

**Figure 1:**
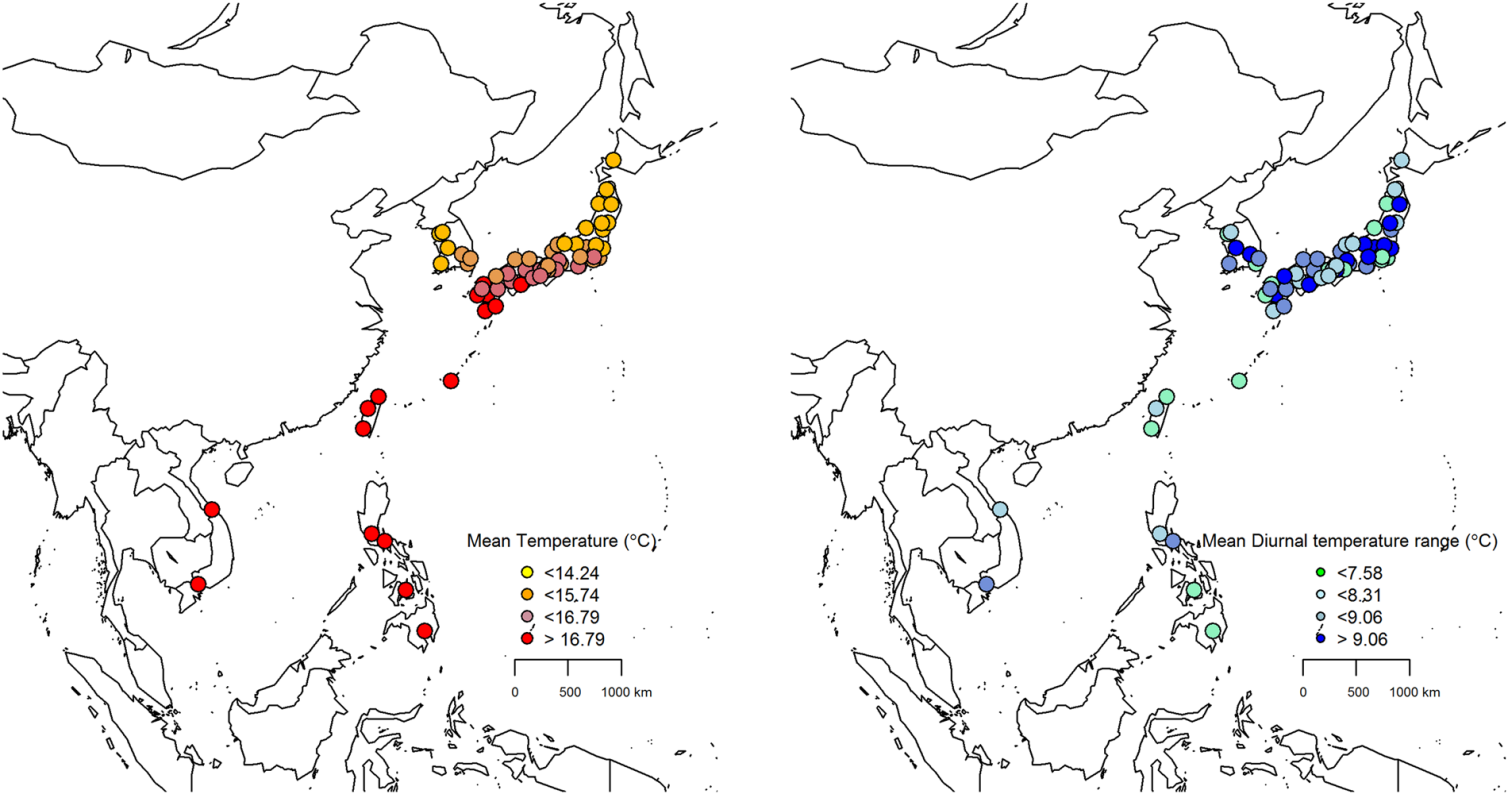
Geographic distributions of mean temperature and diurnal temperature range (DTR) for 63 locations in six East Asian countries/regions included in analysis. The darker colors means the higher mean temperature and diurnal temperature range. Packages “maps” and “mapdata” in R software (3.3.1 version, https://www.r-project.org/) were used.

Figure 1 shows the geographical distributions of the mean temperature and DTR for the 63 locations that were included in this study. We found trends for an association of low-latitude and high-latitude locations with a higher mean temperature and a higher mean DTR, respectively.

Table 1 shows the descriptive statistics for each country. There were 36,089,344 non-accidental deaths, 13,317,378 cardiovascular-related deaths, and 4,608,350 respiratory-related deaths across the five countries/regions. As we expected, each of the five East Asian countries/regions experienced a broad temperature range, such that the country-specific mean varied widely, from 13.72 °C in Korea to 28.06 °C in the Philippines. These temperatures represent the diverse climate conditions of the following regions: areas of northeast Asia (Japan, Korea), southeast subtropics (Chinese Taiwan), and southeast tropics (Vietnam, the Philippines). The average DTR was slightly higher in Northeast Asian countries/regions than in South Asian countries/regions, ranging from 7.10 °C in Chinese Taiwan to 8.41 °C in Japan.

**Table 1:** Descriptive statistics by country/region; SD: Standard deviation.

Country/Region	Number of Locations	Study-Periods	Temperature (°C, SD)	Diurnal temperature range (°C, SD)	Non-accidental Death	Cardiovascular related- Death	Respiratory related- Death
Japan	47	1972–2012	15.08 (8.61)	8.41 (3.30)	33,511,400	12,605,176	4,413,875
South Korea	7	1992–2010	13.72 (9.34)	8.19 (3.28)	1,511,961	435,457	91,135
Chinese Taiwan	3	1994–2007	24.03 (4.72)	7.10 (2.44)	688,394	164,911	63,180
Vietnam	2	2009–2013*	26.91 (3.48)	8.26 (2.61)	102,400	24,433	8,970
The Philippines	4	2003–2010	28.06 (1.43)	7.25 (2.10)	275,189	31,190	31,190
**Total**	63	1972–2013	15.34 (8.76)	8.36 (3.28)	36,089,344	13,317,378	4,608,350

^*^Two cities of Vietnam had different study periods: Ho chi minh city has 2010–2013 and Hue city has 2009–2013 each.

**Figure 2:**
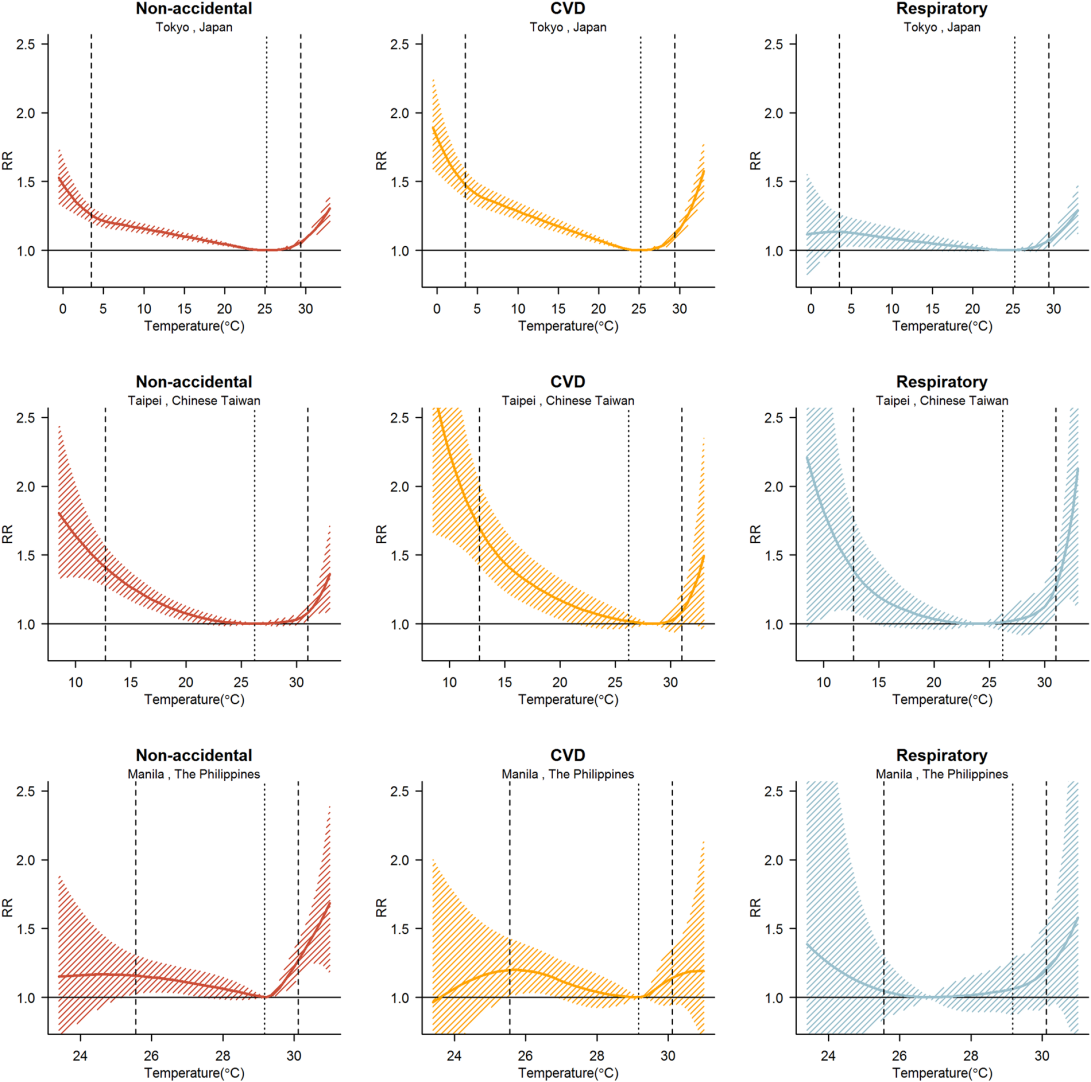
Cumulative exposure-response relations of total (non-accidental), cardiovascular-related disease (CVD), and respiratory-related morality in 63 east-Asia locations: Thick dashed lines are the 2.5th and 97.5th percentiles as cut offs. Light dashed lines are minimum mortality temperatures. Using city-specific distributed lag non-linear model.

Figure 2 shows the overall cumulative exposure–response curves using the best linear unbiased predictions for three of the locations (Tokyo, Taipei, and Manila) that represent each climate condition. For non-accidental mortality, the minimum mortality percentile ranges were at approximately the 90th and 80th percentiles for Tokyo and Manila, whereas Taipei was at approximately the 57th percentile. In Tokyo, the relative risk (RR) increased gradually for cold and hot temperatures (lower and higher than the minimum mortality temperature), and in Taipei, the RR increased more sharply for cold temperatures. However, in Manila, the RR of hot temperatures significantly increased. In Tokyo and Taipei, the RR increased more rapidly for extreme cold of cardiovascular-related mortality than of non-accidental mortality. In addition, the respiratory-related mortality in subtropical and tropical locations had sharply increased RRs for extreme cold and hot temperatures than for other cause-specific mortality. The corresponding temperature–non-accidental mortality curves and minimum mortality percentiles of the 63 locations are reported in the supplementary materials (Supplementary Fig. S1 and Supplementary Table S5, respectively). This tendency similarly appeared for overall regions.

**Figure 3:**
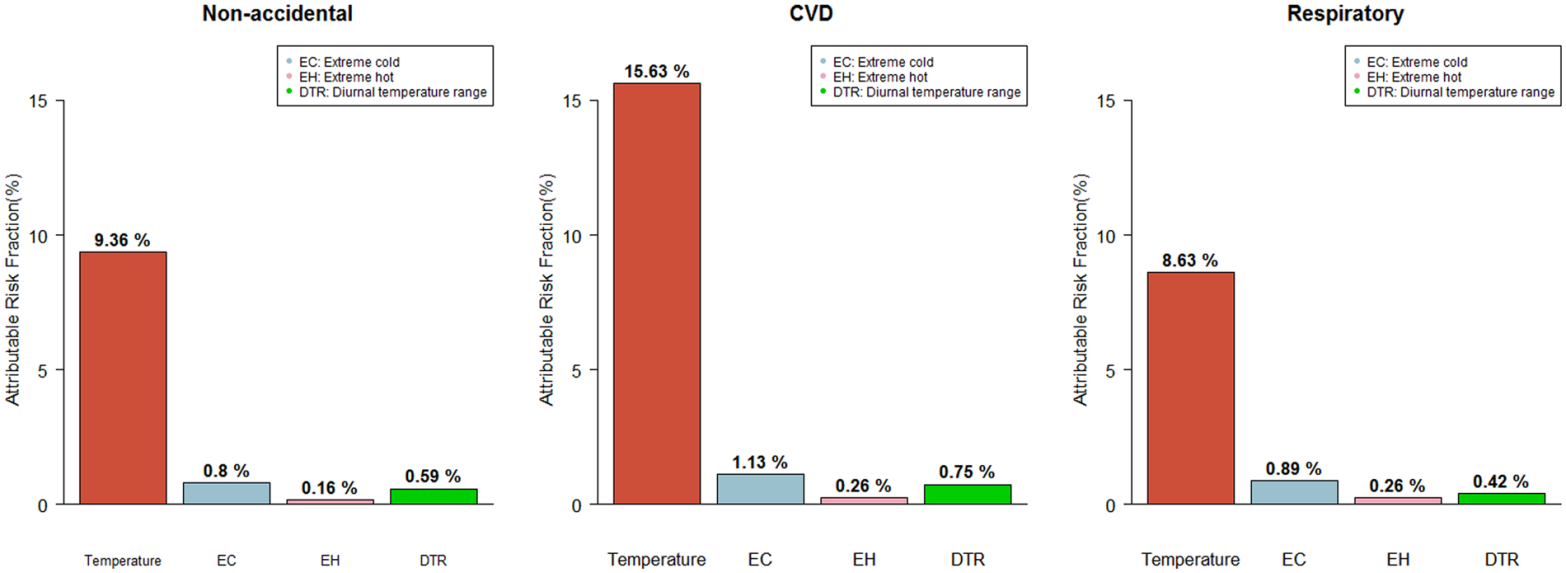
Attributable fractions of cause-specific mortality to overall temperature, extreme cold-hot temperature, and diurnal temperature range. Extreme cold and heat temperature were defined with <2.5^th^ and >97.5^th^ percentiles of temperature distribution.

The main results in Table 2 and Fig. 3 show the estimated attributable fraction by cause-specific mortality; calculations were performed separately for all-season temperature, two extreme temperatures, and DTR by country. Extreme cold and hot temperatures were defined as <2.5th percentile and >97.5th percentile of temperature distribution, respectively. Overall, the total fraction of non-accidental death by temperature was 9.36% (95% empirical confidence interval [eCI]: 8.98–9.69) and varied from 6.37% (95% eCI: 3.60–8.94) in the Philippines to 11.38% (95% eCI: 3.22–18.95) in Vietnam. Moreover, the total attributable fraction was the highest for cardiovascular-related death (15.63%; 95% eCI: 15.04–16.11) and the lowest for respiratory-related death (8.63%; 95% eCI: 6.93–9.39). Specifically, in non-accidental mortality, a relatively high fraction occurred with extreme cold (0.80%; 95% eCI: 0.77–0.83), while extreme hot was responsible for a relatively small fraction (0.16%; 95% eCI: 0.14–0.18).

**Table 2:** Attributable fractions of cause-specific mortality by country/region Empirical confidence interval), DTR: Diurnal temperature range.

		Attributable Mortality Fractions (95% eCI.)											
		Non-accidental				Cardiovascular-related				Respiratory-related			
Country/Region	Periods	Temper-ature	Extreme cold	Extreme hot	DTR	Temper-ature	Extreme cold	Extreme hot	DTR	Temper-ature	Extreme cold	Extreme hot	DTR
Japan	1972–2009	9.38 (9.01, 9.72)	0.82 (0.79, 0.85)	0.15 (0.14, 0.17)	0.58 (0.53, 0.63)	15.8 (15.29, 16.30)	1.15 (1.11, 1.18)	0.26 (0.24, 0.29)	0.73 (0.94, 0.82)	8.2 (7.27, 8.99)	0.9 (0.81, 0.96)	0.23 (0.21, 0.25)	0.43 (0.33, 0.52)
Korea	1992–2010	10.33 (7.86, 12.38)	0.47 (0.29, 0.62)	0.14 (0.06, 0.21)	0.8 (0.33, 1.26)	15.62 (11.70, 18.93)	0.79 (0.53, 1.00)	0.19 (0.05, 0.32)	0.9 (0.02, 1.73)	14.88 (5.77, 21.36)	0.39 (−0.34, 0.82)	0.5 (0.30, 0.66)	0.16 (−0.57, 0.86)
Chinese Taiwan	1994–2009	6.91 (5.41, 8.29)	0.99 (0.75, 1.18)	0.28 (0.16, 0.36)	0.05 (−0.18, 1.12)	11.64 (6.71, 16.05)	1.42 (1.11, 1.64)	0.26 (−0.01, 0.46)	1.21 (−0.15, 2.55)	10.11 (1.69, 16.59)	1.38 (0.85, 1.74)	0.85 (0.60, 1.05)	0 (−1.68, 1.79)
Vietnam	2009–2013	11.38 (3.22, 18.95)	0.13 (−0.37, 0.50)	0.9 (0.44, 1.27)	−1.02 (−5.70, 3.44)	21.38 (8.28, 31.49)	0.39 (−0.39, 0.79)	1.28 (0.50, 1.86)	0.1 (−4.27, 4.02)	30.44 (−456.47, 55.51)	−0.12 (−235.31, 1.20)	0.11 (−1.88, 0.23)	1.62 (−11.32, 12.78)
The Philippines	2006–2010	6.37 (3.60, 8.94)	0.36 (0.02, 0.59)	0.68 (0.52, 0.80)	1.28 (−0.10, 2.48)	2.86 (−4.88, 8.04)	0.01 (−0.71, 0.39)	0.31 (−0.01, 0.59)	0.84 (−1.12, 2.86)	15.16 (4.36, 23.17)	0.67 (−0.08, 1.41)	1.13 (0.74, 1.41)	1.23 (−1.49, 3.68)
**Total**		9.36 (8.98, 9.69)	0.8 (0.77, 0.83)	0.16 (0.14, 0.18)	0.59 (0.53, 0.65)	15.63 (15.04, 16.11)	1.13 (1.08, 1.16)	0.26 (0.24, 0.29)	0.75 (0.65, 0.84)	8.63 (6.93, 9.39)	0.89 (0.18, 0.93)	0.26 (0.23, 0.28)	0.42 (0.31, 0.52)

^*^Model for respiratory mortality in Vietnam could not be converged because of lack of ‘sample size.

^*^Extreme cold and extreme hot temperatures were defined as <2.5^th^ and >97.5^th^ percentiles of temperature distribution, respectively.

DTR had a significant but relatively small effect of 0.59% on non-accidental mortality (95% eCI: 0.53–0.65). Similar results were shown for cardiovascular-related and respiratory-related mortality. The total burden of cardiovascular-related death was 15.63% (95% eCI: 15.04–16.11), and 0.75% (95% eCI: 0.65–0.84) for temperature and DTR, respectively. In the same order, respiratory-related death showed a burden of 8.63% (95% eCI: 6.93-9.39) and 0.42% (95% eCI: 0.31–0.52) for temperature and DTR, respectively. Lag-response plots for DTR are described in Supplementary Fig. 2, and location-specific attributable fractions with three specific mortality are reported in Supplementary Tables S2–S4.

Although the Cochran Q test provides evidence for heterogeneity in all the models, a substantial amount is explained by three predictors (average temperature, temperature range, and country indicators), as indicated by the drop in the I^2^. The meta-regression of the temperature-response relationship had heterogeneity values (I^2^) of 40.8% for non-accidental mortality, 26.8% for cardiovascular-related mortality, and 13.1% for respiratory-related mortality. The meta-regression of the DTR and response relation all had I^2^ values of 1.0%. Although we used all three predictors to modify the exposure–response association, heterogeneities based on single predictors or only the intercept were not significantly different for each model (see Supplementary Table S6).

## Discussion

This study was conducted with the largest dataset to study the attributable fraction of temperature and temperature change on mortality risk. A total of 36,089,344 deaths from five East Asian countries/regions was also included. By analyzing five different countries/regions that have various climate conditions, demographics, and socioeconomic features, our study could provide evidence for an association between temperature variability and death in East Asia. Moreover, by analyzing cause-specific death, we can compare the extent to which the temperature variability indices are attributed to the different causes of death. Our findings show that ambient temperatures and DTR were significantly related to the attributable fractions of total, cardiovascular-related, and respiratory-related mortality in all five countries/regions over the duration of the study periods, and the contribution of the daily mean temperature was much greater than that of the DTR, with total temperature and DTR contributing 9.36% and 0.59% of the risk for non-accidental mortality, respectively. In addition, the influence of ambient temperature and DTR was the highest for cardiovascular-related mortality than for other types of mortality.

Our results are consistent with previous studies, although there were some limitations because the results possibly varied depending on study design, modeling framework, or risk measure. Gasparrini *et al*. reported the attributable all-cause deaths due to ambient temperature in East Asia as follows: 10.12% in Japan, 7.24% in Korea, and 4.75% in Chinese Taiwan^1^. Yang *et al*. described that 17.1% of cardiovascular disease mortality was attributable to ambient temperature in China^19^. Moreover, Yang *et al*. found that ambient temperature was responsible for 14.5% of stroke deaths for 16 large cities in China^20^. Focusing only on the extreme temperature, Gasparrini *et al*. showed the all-cause deaths attributed to extreme temperature in East Asia as follows: extreme cold, 0.77% and extreme heat, 0.18% in Japan, extreme cold, 0.35% and extreme heat, 0.21% in Korea, and extreme cold, 0.71% and extreme heat, 0.25% in Chinese Taiwan^1^. Moreover, similar and consistent tendency-attributable fractions were described, including Hajat and colleagues’ work showing 0.37% to 1.45% of all-cause mortality fractions attributable to heat in three European cities^21^. In addition, the findings of previous studies in Asian countries/regions that focused on effects on mortality of DTR suggested a non-accidental mortality increase of 0.4–1.4%, cardiovascular-related mortality increase of 0.2–1.8%, and respiratory-related mortality increase of 0.7–1.5% per 1 °C incremental increase in DTR^4, 9–11^.

Whereas, in our study, after considering the daily mean temperature effect on mortality, ten units of DTR (°C) was associated with about a 0.5–2% increase in excessive mortality risk. We supposed that previous studies overestimated the relative risk of DTR, because their model strategy did not consider any identifying association issues (both ambient temperature and DTR are derived using daily min and max temperature) arising when temperature indices were considered covariates or confounders during the modeling process. Actually, our results correspond with relatively new research describing excessive DTR mortality risks from −0.29% to 0.4% for six cities in Europe and the United States^17^. However, although the researchers also applied a modeling framework that can adjust for identifying relationships using indicator variables, they could not assess the lag effects of DTR. In contrast, by using a two-step procedure, we ensured that the DTR effects considering lag structure were estimated after removing the overall effects of the daily temperature and seasons^22^.

Numerous assumptions and much of the evidence of underlying medical and biological mechanisms have been reported to define the increased risk of mortality associated with ambient temperature. Substantial evidence from the physiology literature has reported that people have difficulty with acclimatization and thermoregulation to extreme cold and hot temperature^23–25^. Regarding cardiovascular mortality, a high temperature is related to the burden of cardiovascular-related motility. An increase in temperature causes blood vessels to dilate, increasing the cardiac output and risk of decompensating heart failure; it also raises platelet counts, blood viscosity, and cholesterol levels. These influences might cause or trigger death from coronary and cerebral thrombosis^26, 27^. Cold temperature is one of the factors that causes blood vessels to become narrow, increasing blood pressure and heart rate^28^. Increases in blood pressure, fibrinogen concentrations, and blood viscosity, in instances of lower temperature, suggest that cold induces cardiovascular stress and may be prevalent in the entire population^29, 30^. One of the underlying reasons for death due to cardiovascular disease appears to be thrombosis due to hemoconcentration in the cold^29^.

Previous studies identified that the effect of DTR on death independently exist with ambient temperature^4, 10^, and a recent study reported that the variability of temperature is significantly associated with increasing mortality in multi countries, even if daily mean (or maximum and minimum) temperature effect on mortality is also be considered^25^. Our study consistently showed the independent DTR effect with temperature on mortality using daily mean temperature as a confounder variable. Much of the biological evidences suggest that an abrupt change of temperature causes cardiovascular-related and respiratory-related death. Medical studies reported that weather changes might affect the human immune system^27, 31^. In addition, Murayama and Luurila showed that the cardiovascular workload might be increased by sudden temperature changes^32, 33^ and rapid temperature change could also cause the onset of cardiovascular events by affecting workload, heart rate, and oxygen uptake^34^. In respiratory diseases, Graudenz *et al*.^35^ reported that rapid temperature changes may influence inflammatory nasal responses in rhinitis patients and were more strongly connected with elderly asthma patients.

However, the role of temperature in DTR-related mortality is still uncertain. A study in Korea suggested that the effects of DTR increased during warmer seasons^36^, while Chinese studies have reported that DTR has smaller effects on non-accidental mortality during higher temperatures^4, 10^. Furthermore, global warming factors (greenhouse gases, urbanization, and aerosols) have led to decrease in the DTR during recent decades because the nocturnal minimum temperatures have increased faster than the maximum temperatures^37^. Therefore, the effect of decreasing DTR and increasing mean temperature on DTR-mortality should be further studied. Additionally, as a representative indicator of global warming, the effect of heat and cold waves on DTR-mortality also needs to be identified. We expect that in-depth studies will be conducted in various countries or regions that have different climates and alert systems for extreme weather events.

One of the strengths of the study is in its use of advanced statistical approaches, including multivariate random-effect meta-analysis and the distributed non-linear lag model, to estimate the temperature variability-mortality associations and pool effects across cities and countries/regions. Although the Cochran Q test provides evidence for residual heterogeneity in all the models, a substantial amount is explained by three meta-predictors (average temperature, temperature range, and country indicator), as indicated by the higher decrease in the I^2^ statistics when three variables were included in the meta-regression. These methods have advantages in estimating the lag-exposure relation without strong assumptions about the lag structures. In addition, we used a two-step regression to avoid an nonidentifiability^17^ problem between temperature indices and to compare contributions of ambient temperature and DTR, more clearly. One major finding from this study is that ambient temperature accounted for a greater mortality fraction than DTR. Because the attributable fraction considers the distribution of each variable, we can suppose that the attributable fraction is a better measure than RR for comparing contributions of temperature indices. Besides, we also used cause-specific mortalities (cardiovascular-related and respiratory-related mortality) including total mortality and found meaningful associations between temperature variability indices and cause-specific mortalities.

This study also has some limitations. First, we studied slightly different periods in each country, which could induce temporal variability of the estimates. Previous studies reported that temperature variability changed over time, especially DTR, which has decreased worldwide over the last several decades^37–39^. To adjust for the difference of time periods, we estimated the attributable fractions across the same long study period, from 1994 to 2007, of three countries/regions (i.e., Japan, Korea, and Chinese Taiwan) and found that the effect size was slightly attenuated but was not significantly different from the main findings with various study periods (see Supplementary Table S1). Second, we could not adjust for an influenza epidemic as a confounder in the model of respiratory-related death, which would allow for an overestimation or underestimation of attributable fractions. Because we only had an influenza epidemic variable for 10 cities in two countries/regions (Korea and Chinese Taiwan), we did not include influenza in our main results. Instead, we reported the attributable fractions of two countries/regions, adjusting for an influenza epidemic as shown in the supplementary materials, and the results were still robust. Third, ecological variables in this study (across multiple monitors in each city) were used to determine city-specific values, with the assumption of spatial homogeneity for a city^15^. This assumption should be investigated more thoroughly by assessing the spatial patterns of each city. Fourth, the current findings cannot necessarily be interpreted as being representative of other cities and countries/regions with different climates, socioeconomic characteristics, and public health policy. In particular, the Japanese locations (47 prefectures) accounted for most of the locations and periods, potentially allowing for biased results. Therefore, future studies should strive to overcome these limitations by expanding the study populations through the monitoring of other cities and nations. In addition, we could not adjust for the humidity variable because of data limitation. Although such a contribution did not substantially affect the temperature-related mortality association, the humidity needs to be considered in future study as a confounder.

In summary, this study found a substantial impact on temperature and DTR on mortality in five Asian countries/regions. Overall, the ambient temperature and DTR are significantly associated with increases in mortality risk, and the ambient temperature on mortality as a major role. Moreover, cardiovascular-related mortality is the most susceptible to temperature and DTR than total/respiratory-related mortality. We hope our results can also support public health offices or researchers actively engaged in studies that consider the risk and health burden of ambient temperature and DTR on mortality.

## Methods

This study included 63 cities; 3 cities in Chinese Taiwan for 1994–2007, 7 cities in Korea for 1992–2010, 47 prefectures in Japan for 1972–2009, 2 cities in Vietnam 2009–2013 and 4 cities in Philippines for 2003–2010. Daily mortality excluded accidental causes (hereafter referred to as a non-accidental mortality). Two specific causes (cardiovascular- and respiratory-related) of daily mortality were considered. Based on the International Classification of Diseases Revision 10 (ICD-10), non-accidental mortality as ICD-10 (A00-R99) cardiovascular-related mortality was defined as ICD-10 (I00-I99), and respiratory-related mortality was defined as ICD-10 (J00-J99). Only in Philippines, we used all-cause mortality instead of non-accidental mortality, because non-accidental mortality was not available. Weather variables included the daily mean, maximum, and minimum temperature (°C). Mortality counts and weather data for each city were obtained from different sources for some cities; air pollution data were only available for some years of the study periods^15^. And source of the weather data and study periods are shown in supplementary materials (see Supplementary Table S7). A daily categorical variable for influenza epidemics was created assigning a value of 1 if the moving average of the daily number of influenza deaths per 1000 all-cause deaths over the previous week was ≥1; otherwise, a value of 0 was assigned. This way to adjust the influenza effect was also used in previous research^15^.

We used a two-step regression approach analysis. In the first step, we fitted distributed lag non-linear model to consider the non-linear relationship between temperature-mortality and the non-linear delayed effect of temperature on mortality simultaneously, with every city with day of week, seasonal and long term trend. Then we fitted a model to estimate extra effects of DTR. We used this two-step regression to ensure that the DTR effects were estimated after removing the overall effects of temperature and season. We used the following quasi-Poisson regression model for time-series analysis in each city firstly,

1 $$\begin{array}{c}{Y}_{t}\sim quasiPoisson({\mu }_{t}),\,{\rm{t}}=1,\ldots T\\ \mathrm{log}({\mu }_{t})={\beta }_{0}+s(TEM{P}_{t})+factor(DO{W}_{t})+ns(TIM{E}_{t},df=8\,per\,yr)\end{array}$$

where Y_t_ = death count on day *t*, μ_t_ = expected death count on day *t*, *β*_0_ = intercept of the model, s(*TEMP*_*t*_) = basis of ambient temperature on day *t*. The quasi-Poisson likelihood was used to consider the over-dispersion^1, 3^. Specifically, we used the exposure-response curve with quadratic B-spline with three internal knots placed at 10^th^, 75^th^, and 90^th^ percentiles of location-specific temperature distributions, and the lag-response curve with a natural cubic spline with an intercept and three internal knots placed at equally spaced values in the log scale. This basis is referenced from previous study^1^. We extended the lag period to 28 days. *DOW*_*t*_ = categorical variable for day of week on day *t*, *TIME*_*t*_ = time on day *t* using 8 degrees of freedom(df) per year. In order to estimate the effects of DTR (difference between daily maximum and minimum temperature) in each city we used,

2 $$\begin{array}{c}{y}_{t} \sim quasiPoisson({{\mu }_{t}}^{\ast }),t=1,\ldots {\rm{T}}\\ \mathrm{log}({{\mu }_{t}}^{\ast })=\,\mathrm{log}({\hat{\mu }}_{t})+DT{R}_{t}\end{array}$$

where ${{\mu }_{t}}^{\ast }$ is the predicted values from equation (1). We assumed the association between mortality and DTR as a linear, referenced from previous studies^9, 10, 36^, so we applied an cross-basis function for DTR with exposure-response is linear and the lag-response curve with a natural cubic spline with an intercept and two internal knots placed at equally spaced values in the log scale. We extended the lag period for DTR to 14 days. This choice of lag days was motivated by previous studies reporting a delayed effect of DTR^2, 4, 10^. All processes of two step analysis were accomplished using R package *dlnm* ^40^. Sensitivity analyses were performed to test the consistency of the results with various modeling choices such as various lag days of temperature indices, degrees of freedom for indices and time trend, and adjustments for humidity and an influenza epidemic. The supplementary materials includes sensitivity analysis results (see Supplementary Table S1). The results of these sensitivity analyses indicate that our results are not dependent on the modeling assumptions.

Before we pooled the estimated city-specific results, we reduced the association to the overall temperature-mortality and DTR-mortality associations, cumulating the risk during lag periods by summing estimates of all lag periods from equations (1) and (2)^41^. These associations were reduced to two summaries: the overall cumulative exposure-response relation and the lag-response relation specific to the 2.5^th^ and 97.5^th^ percentiles of temperature distributions and 99^th^ percentiles of DTR distributions. This definitions have been previously reported^1, 41, 42^. After reducing parameters, we pooled the estimated city-specific overall cumulative exposure-response associations and the city-specific lag-exposure associations using a multivariate meta-analysis. We obtained city-specific average temperature, temperature range, and country indicators as meta-predictors in a multivariate meta-regression. We tested heterogeneity through a multivariate extension if indicated by the Cochran Q statistic and I^2^ index^42, 43^ and these results are described in Supplementary Table S6.

We used the fitted random-effect multivariate meta-regression models to derive the best linear unbiased prediction (BLUP) for the exposure-response and lag-response association respectively in each city. This process allows analysis of cities with a small population, small number of death counts, or short study periods, generally described by imprecise estimates^1, 44^. The second-stage analysis was accomplished with the R package *mvmeta* ^42^. City-specific attributable fractions and minimum mortality temperature of each city were estimated from second-stage analysis are reported in supplementary materials. Minimum mortality temperature (an estimated temperature at which mortality was the lowest by the BLUP), used it the reference to calculate relative risks and attributable risk fraction for lag days.

The total attributable number for three different causes of death associated with temperature indices was obtained by the sum of the contributions from all the days of study, and we obtained the attributable fraction using the total attributable number ratio corresponding with the total number of deaths^1^. The fraction attributable to indices was calculated by summing the subsets corresponding to the days of minimum temperature to 0.25% (extreme cold), 97.5% to maximum temperature (extreme hot) and minimum to maximum DTR to reflect the total effect. We calculated the empirical confidence intervals (eCI.)s using Monte Carlo simulations, with an assumption of multivariate normal distribution of the reduced BLUP coefficient. All these processes for analyzing attributable risk also have been used in previous studies^1, 45^.

## Electronic supplementary material

Supplementary Information
