# Supplementary material for: An Investigation on Attributes of Ambient Temperature and Diurnal Temperature Range on Mortality in Five East-Asian Countries
Source: Sci Rep. 2017 Aug 31;7:10207. doi: 10.1038/s41598-017-10433-8 (PMC5579298; doi:10.1038/s41598-017-10433-8)
Supplement: Supplementary file 1 — Supplementary Information [file 41598_2017_10433_MOESM1_ESM.pdf]

Supplementary Information.

Title: An Investigation on Attributes of Ambient Temperature and Diurnal Temperature Range on Mortality in Five East-Asian Countries

Author Affiliation:

Whan-Hee Lee[1], Youn-Hee Lim[2,3], Tran Ngoc Dang[4], Xerxes Seposo[4], Yasushi Honda[4], Yue-Liang Leon Guo[5], Hye-Min Jang[6], and Ho Kim[1]

[1]Graduated School of Public Health, Seoul National University, Seoul, Republic of Korea. [2] Institute of Environmental Medicine, Seoul National University of Medical Research Center, Seoul National University, Republic of Korea. [3]Environmental Health Center, Seoul National University College of Medicine, Republic of Korea. [4]Faculty of Health and Sports Sciences, University of Tsukuba, Tsukuba, Japan. [5]Department of Environmental and Occupational Medicine, National Taiwan University, Taipei, Taiwan. [6]Department of Statistics, Ewha Womans' University, Seoul, Republic of Korea.

Corresponding Author: Ho Kim, Graduated School of Public Health, Seoul National University, 1 Gwanak-ro, Gwanak-gu, Seoul 151-742, Republic of Korea. Telephone: (82) 2 880-2702 E-mail:hokim@snu.ac.kr

Supplementary Table S1. Estimated on the fraction (%) attributable to ambient temperature and diurnal temperature range (DTR) by various model choices as a sensitivity analysis.

**Total death**	Temperature (%)	Extreme Cold (%)	Extreme Heat (%)	Diurnal Temperature Range (%)
Main Model	9.36	0.80	0.16	0.59
Knots for temperature-mortality: 10th, 50th, and 90th	8.92	0.78	0.14	0.59
Knots for temperature-mortality: 10th, 25th, 75th, and 90th	9.09	0.78	0.16	0.63
Cubic B-spline for temperature-mortality	9.36	0.80	0.16	0.59
Df for temperature lag response: 6	9.15	0.79	0.16	0.58
Df for DTR lag response: 5	9.36	0.80	0.16	0.58
Temperature lag periods: 14 days	10.53	0.77	0.24	0.59
Temperature lag periods: 21 days	10.10	0.82	0.20	0.54
DTR lag: 7 days	9.36	0.80	0.16	0.97
Df/year for seasonal control: 6	7.25	0.76	0.23	0.77
Df/year for seasonal control: 7	9.38	0.81	0.16	0.67
Df/year for seasonal control: 10	6.52	0.63	0.18	0.52
Only three countries (Japan, Korea and Chinese Taiwan) with periods 2004 to 2007	8.30	0.71	0.15	0.47
Add influenza epidemic (10 cities of Korea and Chinese Taiwan)	8.66	0.56	0.20	0.85
Add humidity (Japan, Korea, and Chinese Taiwan)	10.24	0.83	0.21	0.86

**Cardiovascular-related death**	Temperature (%)	Extreme Cold (%)	Extreme Heat (%)	Diurnal Temperature Range (%)
Main Model	15.36	1.13	0.26	0.75
Knots for temperature-mortality: 10th, 50th, and 90th	14.99	1.00	0.28	0.77
Knots for temperature-mortality: 10th, 25th, 75th, and 90th	14.93	1.02	0.25	0.71
Cubic B-spline for temperature-mortality	16.21	1.07	0.30	0.74
Df for temperature lag response: 6	16.08	1.06	0.30	0.73
Df for DTR lag response: 5	16.21	1.07	0.30	0.74
Temperature lag periods: 14 days	17.49	1.12	0.40	0.74
Temperature lag periods: 21 days	17.16	1.13	0.34	0.71
DTR lag: 7 days	16.21	1.07	0.30	1.08
Df/year for seasonal control: 6	12.09	0.96	0.29	1.22
Df/year for seasonal control: 7	16.39	1.29	0.23	0.84
Df/year for seasonal control: 10	11.42	0.91	0.27	0.99
Minimum mortality temperature of non-accidental mortality	17.28	1.32	0.27	0.78
Only three countries (Japan, Korea and Chinese Taiwan) with periods 2004 to 2007	13.89	1.03	2.63	0.85
Add influenza epidemic (10 cities of Korea and Chinese Taiwan)	11.16	0.84	0.16	0.96
Add humidity (Japan, Korea, and Chinese Taiwan)	17.32	1.32	0.27	0.78

**Respiratory-related death**	Temperature (%)	Extreme Cold (%)	Extreme Heat (%)	Diurnal Temperature Range (%)
Main Model	8.63	0.89	0.26	0.42
Knots for temperature-mortality: 10th, 50th, and 90th	7.58	0.87	0.28	0.42
Knots for temperature-mortality: 10th, 25th, 75th, and 90th	Not converged	Not converged	Not converged	Not converged
Cubic B-spline for temperature-mortality	8.60	0.88	0.26	0.43
Df for temperature lag response: 6	8.60	0.88	0.26	0.43
Df for DTR lag response: 5	8.60	0.88	0.26	0.42

Temperature lag periods: 14 days	9.55	0.90	0.30	0.52
Temperature lag periods: 21 days	8.92	0.89	0.28	0.43
DTR lag: 7 days	8.60	0.88	0.26	0.49
Df/year for seasonal control: 6	9.53	0.89	0.29	0.71
Df/year for seasonal control: 7	7.04	0.86	0.22	0.48
Df/year for seasonal control: 10	8.50	0.88	0.26	0.44
Minimum mortality temperature of non-accidental mortality	8.18	0.81	0.30	0.39
Only three countries (Japan, Korea and Chinese Taiwan) with periods 2004 to 2007	6.15	0.55	0.20	0.60
Add influenza epidemic (10 cities of Korea and Chinese Taiwan)	15.20	0.62	0.73	0.48
Add humidity (Japan, Korea, and Chinese Taiwan)	8.50	0.84	0.33	0.54

Supplementary Table S2. Attributable (non-accidental) mortality fractions (AF, %) by city, with 95% empirical confidence intervals.

		Attributable Fractions for Total Mortality (%)											
**Country /Region**	**City**	**Temperature AF (95% eCI)**			**Extreme Cold AF (95% eCI)**			**Extreme Hot AF (95% eCI)**			**Diurnal Temperature Range AF (95% eCI)**		
Japan	Aichi	10.47	8.67	12.13	0.88	0.75	1.00	0.24	0.15	0.32	0.63	0.40	0.86
	Akita	11.61	9.32	13.76	0.79	0.54	1.03	0.26	0.16	0.36	0.47	0.20	0.76
	Aomori	12.60	9.82	15.27	0.73	0.47	0.95	0.37	0.25	0.49	0.47	0.09	0.86
	Chiba	9.48	7.83	11.12	0.76	0.61	0.92	0.11	0.03	0.19	0.58	0.33	0.81
	Ehime	8.65	6.59	10.48	0.93	0.76	1.08	0.11	0.00	0.20	0.60	0.36	0.83
	Fukushima	11.65	9.51	13.68	0.90	0.67	1.11	0.29	0.17	0.40	0.65	0.37	0.95
	Fukui	10.52	8.48	12.64	0.96	0.71	1.18	0.21	0.09	0.32	0.62	0.42	0.82
	Fukuoka	7.48	5.38	9.56	0.84	0.70	0.96	0.10	-0.01	0.20	0.52	0.24	0.75
	Gifu	9.42	7.39	11.41	0.96	0.77	1.13	0.16	0.05	0.26	0.64	0.39	0.89
	Gunma	9.62	7.81	11.33	0.82	0.64	1.00	0.17	0.05	0.27	0.67	0.46	0.89
	Hokkaido	8.55	5.30	11.58	0.43	0.19	0.62	0.18	0.07	0.28	0.33	-0.20	0.88
	Hiroshima	8.37	5.63	10.89	0.82	0.66	0.98	0.17	0.03	0.28	0.50	0.08	0.95
	Hyogo	6.81	4.82	8.54	0.82	0.69	0.94	0.03	-0.06	0.13	0.47	0.24	0.72
	Ibaraki	11.16	9.11	13.23	0.93	0.72	1.11	0.15	0.05	0.24	0.74	0.40	1.11
	Ishikawa	10.16	8.42	11.88	0.82	0.60	1.02	0.22	0.11	0.32	0.53	0.36	0.72
	Iwate	12.21	9.26	15.24	0.65	0.35	0.93	0.30	0.18	0.42	0.59	0.07	1.12
	Kagawa	8.93	6.71	11.19	0.90	0.73	1.07	0.14	0.00	0.27	0.61	0.36	0.84
	Kanagawa	9.21	7.08	11.42	0.61	0.43	0.78	0.13	0.02	0.24	0.62	0.24	0.97
	Kagoshima	8.80	6.70	10.72	1.26	1.09	1.40	0.15	0.02	0.27	0.64	0.28	0.98
	Kochi	8.00	5.93	9.86	1.13	0.93	1.31	0.03	-0.05	0.12	0.71	0.41	1.01
	Kumamoto	8.38	6.49	10.22	1.12	0.96	1.27	0.06	-0.06	0.17	0.72	0.41	1.03
	Kyoto	9.01	6.85	10.92	0.83	0.66	0.98	0.14	0.05	0.24	0.63	0.37	0.89
	Mie	9.39	7.20	11.52	1.04	0.85	1.19	0.10	-0.01	0.20	0.53	0.33	0.74
	Miyagi	10.85	8.57	13.07	0.86	0.64	1.05	0.13	0.04	0.23	0.57	0.25	0.87
	Miyazaki	9.29	7.38	11.34	1.21	1.04	1.38	0.11	0.01	0.21	0.72	0.37	1.05
	Nagano	11.65	9.38	13.85	0.87	0.64	1.06	0.22	0.13	0.31	0.64	0.29	0.98
	Nagasaki	7.87	5.71	10.18	0.98	0.80	1.14	0.08	-0.02	0.18	0.52	0.29	0.76
	Nara	10.20	8.23	11.91	0.87	0.68	1.05	0.12	0.03	0.20	0.72	0.49	0.96
	Niigata	11.31	9.64	12.95	0.82	0.66	0.97	0.26	0.17	0.34	0.47	0.31	0.65
	Oita	8.27	6.21	10.18	0.97	0.80	1.13	0.08	-0.02	0.17	0.64	0.40	0.89
	Okayama	8.22	5.97	10.43	0.97	0.80	1.14	0.12	0.00	0.24	0.59	0.25	0.95
	Okinawa	7.95	2.43	13.27	1.10	0.79	1.36	0.00	-0.01	0.01	0.53	0.00	1.06
	Osaka	8.97	7.11	10.67	0.81	0.67	0.93	0.14	0.06	0.22	0.55	0.28	0.77
	Saga	8.70	6.15	11.12	1.05	0.86	1.23	0.11	0.00	0.23	0.64	0.34	0.96
	Saitama	10.39	8.67	11.91	0.81	0.68	0.94	0.20	0.12	0.28	0.69	0.45	0.91

	Shiga	11.09	9.07	13.12	0.98	0.76	1.18	0.16	0.04	0.27	0.56	0.35	0.81
	Shimane	8.72	6.42	10.83	0.90	0.69	1.10	0.13	0.03	0.23	0.53	0.29	0.77
	Shizuoka	9.67	7.78	11.46	0.88	0.71	1.04	0.12	0.04	0.20	0.71	0.39	1.03
	Tokushima	7.72	5.03	10.10	0.89	0.71	1.06	0.08	-0.03	0.19	0.50	0.22	0.77
	Tochigi	11.31	9.15	13.36	0.93	0.75	1.11	0.19	0.09	0.29	0.75	0.44	1.04
	Tokyo	8.72	7.13	10.32	0.57	0.43	0.71	0.14	0.07	0.22	0.54	0.33	0.75
	Tottori	9.13	6.91	11.30	0.95	0.75	1.14	0.17	0.05	0.29	0.58	0.28	0.89
	Toyama	8.88	6.60	11.09	0.65	0.42	0.88	0.16	0.06	0.26	0.51	0.26	0.76
	Wakayama	8.69	6.44	11.05	0.90	0.71	1.09	0.09	-0.01	0.19	0.58	0.34	0.80
	Yamagata	10.83	8.40	13.15	0.62	0.34	0.87	0.26	0.15	0.36	0.58	0.26	0.92
	Yamaguchi	9.07	7.06	10.96	0.90	0.75	1.04	0.17	0.06	0.28	0.68	0.39	0.95
	Yamanashi	10.18	8.11	12.08	0.92	0.69	1.12	0.14	0.03	0.23	0.78	0.52	1.02
Korea	Busan	11.03	5.77	15.94	0.50	0.08	0.86	0.08	-0.13	0.27	0.89	-0.04	1.85
	Daegu	9.90	5.27	14.19	0.31	-0.13	0.69	0.12	-0.06	0.29	1.05	-0.02	2.02
	Daejeon	11.02	6.81	15.04	0.53	0.13	0.88	0.17	0.02	0.31	0.97	0.01	1.93
	Gwangju	11.25	6.21	15.94	0.38	-0.05	0.74	0.16	-0.01	0.33	1.04	0.01	2.07
	Incheon	9.75	5.11	14.00	0.62	0.29	0.93	0.09	-0.04	0.22	0.68	-0.10	1.45
	Seoul	9.90	5.04	14.03	0.45	0.13	0.76	0.17	0.02	0.30	0.65	-0.34	1.58
	Ulsan	12.06	6.51	17.48	0.55	0.07	0.93	0.20	-0.05	0.42	1.05	0.01	2.14
Chinese Taiwan	Kaohsiung	6.21	3.99	8.30	0.97	0.59	1.25	0.31	0.11	0.48	0.51	-0.54	1.54
	Taipei	7.55	4.98	9.67	1.02	0.64	1.33	0.26	0.09	0.42	0.46	-0.59	1.49
	Taichung	6.25	4.06	8.30	0.95	0.55	1.28	0.24	0.07	0.39	0.58	-0.74	1.81
Vietnam	Ho Chi Minh	11.42	2.74	19.35	0.09	-0.44	0.48	0.91	0.41	1.30	-0.99	-6.02	3.81
	Hue	10.58	1.94	16.84	0.80	-1.10	1.77	0.79	0.31	1.18	-1.59	-8.32	4.51
The Philippines	Cebu	6.07	0.68	10.71	0.43	-0.02	0.80	0.63	0.37	0.86	1.08	-1.05	3.13
	Davao	6.74	1.05	12.09	0.26	-0.20	0.60	0.81	0.44	1.08	1.08	-0.97	2.94
	Manila	6.27	0.51	11.57	0.19	-0.60	0.71	0.70	0.39	0.96	1.41	-0.96	3.98
	Quezon	6.53	1.24	11.43	0.41	-0.37	0.90	0.65	0.37	0.88	1.52	-1.28	4.08

Supplementary Table S3. Attributable (cardiovascular-related) mortality fractions (AF, %) by city, with 95% empirical confidence intervals.

		Attributable Fractions for Cardiovascular Mortality (%)											
Country /Region	City	Temperature AF (95% eCI)			Extreme Cold AF (95% eCI)			Extreme Hot AF (95% eCI)			Diurnal Temperature Range AF (95% eCI)		
Japan	Aichi	16.22	14.11	18.39	1.18	1.01	1.34	0.41	0.27	0.53	0.79	0.43	1.15
	Akita	17.65	13.92	20.80	1.13	0.78	1.41	0.37	0.21	0.52	0.62	0.19	1.07
	Aomori	18.27	14.33	22.50	1.01	0.64	1.29	0.50	0.31	0.68	0.63	0.04	1.20
	Chiba	16.18	13.40	18.63	1.07	0.87	1.26	0.22	0.07	0.35	0.72	0.31	1.13
	Ehime	15.60	12.50	18.43	1.25	1.03	1.47	0.21	0.05	0.36	0.74	0.32	1.13
	Fukushima	18.87	15.75	21.87	1.34	1.10	1.56	0.34	0.17	0.50	0.84	0.36	1.33
	Fukui	18.50	15.17	21.49	1.44	1.16	1.68	0.30	0.12	0.46	0.78	0.46	1.13
	Fukuoka	13.98	11.23	16.61	1.15	0.96	1.32	0.22	0.06	0.39	0.64	0.19	1.11
	Gifu	15.91	13.30	18.19	1.27	1.07	1.48	0.33	0.15	0.47	0.80	0.41	1.24
	Gunma	16.59	13.86	19.58	1.18	0.94	1.40	0.30	0.14	0.46	0.85	0.48	1.17
	Hokkaido	13.17	8.61	17.46	0.61	0.28	0.87	0.35	0.19	0.49	0.47	-0.40	1.32
	Hiroshima	14.64	11.05	17.91	1.19	0.96	1.40	0.27	0.08	0.45	0.63	-0.13	1.40
	Hyogo	14.35	11.19	17.23	1.21	1.03	1.38	0.12	-0.03	0.27	0.58	0.18	0.97
	Ibaraki	18.24	15.06	21.50	1.25	1.00	1.46	0.22	0.07	0.36	0.93	0.30	1.56
	Ishikawa	16.76	13.59	19.45	1.21	0.94	1.44	0.33	0.16	0.48	0.68	0.39	0.95
	Iwate	17.74	13.22	21.98	0.96	0.55	1.28	0.42	0.23	0.59	0.79	-0.07	1.57
	Kagawa	16.16	12.98	19.14	1.22	0.96	1.43	0.29	0.07	0.46	0.76	0.32	1.19
	Kanagawa	15.13	11.55	18.81	0.85	0.58	1.09	0.32	0.15	0.49	0.77	0.16	1.41
	Kagoshima	15.51	12.68	18.10	1.57	1.34	1.75	0.22	-0.03	0.42	0.78	0.16	1.34
	Kochi	15.70	12.59	18.44	1.50	1.26	1.71	0.11	-0.02	0.23	0.87	0.34	1.37
	Kumamoto	15.50	12.68	18.18	1.45	1.22	1.64	0.16	-0.04	0.35	0.89	0.36	1.38
	Kyoto	15.95	12.86	18.60	1.21	0.99	1.40	0.24	0.09	0.37	0.78	0.37	1.22
	Mie	17.32	14.68	19.76	1.42	1.20	1.59	0.21	0.07	0.36	0.66	0.30	1.00
	Miyagi	18.66	15.21	21.79	1.24	0.95	1.47	0.22	0.07	0.36	0.73	0.19	1.24
	Miyazaki	16.00	12.85	18.85	1.49	1.23	1.70	0.17	0.01	0.32	0.88	0.30	1.41
	Nagano	17.10	13.35	20.64	1.10	0.82	1.36	0.33	0.19	0.47	0.84	0.28	1.39
	Nagasaki	13.97	11.07	16.65	1.33	1.11	1.54	0.11	-0.04	0.26	0.64	0.23	1.04
	Nara	17.90	14.95	20.79	1.22	0.99	1.42	0.19	0.05	0.34	0.91	0.50	1.33
	Niigata	17.78	15.19	20.25	1.17	0.95	1.38	0.40	0.26	0.52	0.60	0.33	0.88
	Oita	15.59	12.50	18.49	1.30	1.06	1.51	0.15	0.00	0.31	0.79	0.38	1.21
	Okayama	15.55	12.15	18.73	1.34	1.13	1.54	0.19	-0.03	0.36	0.74	0.20	1.30
	Okinawa	13.97	7.82	19.29	1.34	0.92	1.65	0.07	-0.33	0.39	0.62	-0.30	1.52
	Osaka	14.42	12.29	16.80	1.10	0.94	1.27	0.24	0.10	0.38	0.68	0.27	1.08
	Saga	15.98	12.66	19.04	1.48	1.23	1.70	0.16	-0.01	0.32	0.80	0.26	1.33
	Saitama	16.97	14.57	19.34	1.11	0.92	1.27	0.34	0.21	0.45	0.87	0.45	1.26

	Shiga	19.67	16.19	22.96	1.41	1.14	1.63	0.21	0.02	0.38	0.71	0.30	1.10
	Shimane	15.83	12.69	19.00	1.30	1.01	1.52	0.25	0.10	0.40	0.67	0.25	1.07
	Shizuoka	15.98	13.00	18.71	1.26	1.04	1.47	0.14	0.01	0.25	0.87	0.33	1.40
	Tokushima	14.85	11.46	17.91	1.27	1.03	1.49	0.17	-0.01	0.32	0.62	0.17	1.08
	Tochigi	18.94	15.86	21.82	1.28	1.04	1.48	0.34	0.18	0.48	0.95	0.41	1.43
	Tokyo	14.18	11.88	16.15	0.90	0.71	1.07	0.29	0.17	0.40	0.67	0.34	1.02
	Tottori	15.66	12.20	18.60	1.26	1.00	1.49	0.21	0.02	0.41	0.73	0.21	1.27
	Toyama	14.92	11.90	17.73	1.13	0.85	1.37	0.23	0.08	0.37	0.65	0.25	1.03
	Wakayama	15.54	12.42	18.31	1.32	1.06	1.52	0.14	0.01	0.29	0.71	0.32	1.12
	Yamagata	16.22	12.72	19.72	1.00	0.66	1.29	0.36	0.19	0.50	0.76	0.23	1.29
	Yamaguchi	16.59	13.55	19.41	1.32	1.12	1.49	0.21	0.04	0.37	0.86	0.40	1.35
	Yamanashi	16.80	13.80	19.77	1.21	0.91	1.45	0.27	0.14	0.41	0.99	0.57	1.40
Korea	Busan	17.61	8.60	25.66	0.72	0.07	1.19	0.19	-0.22	0.51	1.00	-0.58	2.52
	Daegu	15.44	7.56	22.10	0.73	0.07	1.22	0.19	-0.11	0.47	1.18	-0.61	3.03
	Daejeon	15.84	8.02	22.32	0.81	0.29	1.23	0.18	-0.05	0.39	1.09	-0.87	2.82
	Gwangju	16.30	8.16	23.69	0.66	-0.01	1.13	0.24	-0.05	0.48	1.17	-0.75	3.00
	Incheon	15.53	8.12	21.96	0.87	0.36	1.24	0.13	-0.09	0.34	0.77	-0.62	2.14
	Seoul	14.44	7.06	21.00	0.85	0.35	1.21	0.19	-0.09	0.41	0.72	-1.06	2.41
	Ulsan	17.85	9.53	25.28	0.73	0.11	1.23	0.28	-0.16	0.64	1.19	-0.60	3.05
Chinese Taiwan	Kaohsiung	10.34	2.47	17.00	1.32	0.82	1.69	0.34	-0.10	0.72	1.17	-0.64	3.24
	Taipei	12.50	5.43	19.50	1.50	1.05	1.84	0.21	-0.19	0.55	1.14	-0.86	3.17
	Taichung	11.22	3.29	17.88	1.33	0.73	1.76	0.29	-0.06	0.62	1.41	-1.19	3.87
Vietnam	Ho Chi Minh	21.43	8.15	31.20	0.32	-0.41	0.77	1.28	0.51	1.80	0.12	-4.31	4.21
	Hue	20.66	-11.75	43.38	1.59	-0.43	2.37	1.32	0.24	1.94	-0.20	-5.19	5.02
The Philippines	Cebu	2.61	-5.30	9.92	0.06	-0.95	0.68	0.33	-0.23	0.77	0.69	-2.41	3.69
	Davao	1.96	-6.19	9.56	-0.01	-0.81	0.57	0.34	-0.39	0.90	0.71	-2.43	3.92
	Manila	6.20	-33.23	34.05	-0.03	-0.28	0.03	0.38	-0.92	1.15	0.95	-2.70	4.83
	Quezon	1.94	-4.26	7.50	-0.02	-1.75	0.84	0.25	-0.32	0.68	0.99	-3.77	4.93

Supplementary Table S4. Attributable (respiratory-related) mortality fractions (AF, %) by city, with 95% empirical confidence intervals.

		**Attributable Fractions for Respiratory Mortality (%)**											
**Country /Region**	**City**	**Temperature AF (95% eCI)**			**Extreme Cold AF (95% eCI)**			**Extreme Hot AF (95% eCI)**			**Diurnal Temperature Range AF (95% eCI)**		
Japan	Aichi	5.42	1.75	8.87	0.83	0.45	1.14	0.20	0.10	0.31	0.44	0.05	0.79
	Akita	13.21	7.12	18.32	1.01	0.41	1.44	0.33	0.20	0.43	0.42	-0.12	0.92
	Aomori	12.26	4.52	18.40	0.55	-0.28	1.08	0.45	0.29	0.61	0.45	-0.24	1.13
	Chiba	8.71	4.22	13.16	0.87	0.43	1.18	0.17	0.09	0.25	0.50	0.07	0.93
	Ehime	6.55	3.06	9.66	1.00	0.62	1.33	0.21	0.07	0.33	0.41	0.01	0.81
	Fukushima	11.93	6.28	16.72	1.00	0.42	1.43	0.35	0.20	0.49	0.57	0.03	1.09
	Fukui	8.42	3.81	12.71	1.11	0.59	1.53	0.29	0.18	0.40	0.48	0.12	0.84
	Fukuoka	6.47	3.88	8.79	1.09	0.80	1.36	0.26	0.09	0.42	0.29	-0.17	0.72
	Gifu	7.91	2.89	12.33	1.13	0.67	1.49	0.20	0.11	0.29	0.42	0.01	0.81
	Gunma	7.85	1.25	13.23	0.87	0.45	1.24	0.22	0.15	0.29	0.49	0.13	0.85
	Hokkaido	10.62	2.05	18.27	0.50	-0.05	0.95	0.33	0.16	0.48	0.16	-0.93	1.17
	Hiroshima	6.00	-0.83	12.27	0.82	0.36	1.18	0.22	0.05	0.38	0.17	-0.61	0.90
	Hyogo	6.65	3.08	9.87	0.96	0.61	1.27	0.23	0.08	0.37	0.26	-0.15	0.67
	Ibaraki	10.89	4.83	15.87	1.02	0.51	1.39	0.31	0.19	0.42	0.69	-0.03	1.32
	Ishikawa	7.22	1.91	12.14	0.82	0.29	1.22	0.21	0.14	0.28	0.40	0.11	0.68
	Iwate	14.02	5.98	20.78	0.73	-0.13	1.30	0.46	0.26	0.62	0.59	-0.33	1.49
	Kagawa	9.39	4.44	13.58	1.15	0.78	1.46	0.24	0.10	0.37	0.39	-0.03	0.77
	Kanagawa	9.81	3.75	15.07	0.75	0.29	1.12	0.32	0.14	0.49	0.61	-0.08	1.28
	Kagoshima	9.16	6.47	11.58	1.61	1.29	1.87	0.30	0.09	0.49	0.42	-0.16	0.99
	Kochi	6.89	2.25	10.77	1.07	0.51	1.48	0.13	0.04	0.23	0.49	0.02	1.01
	Kumamoto	8.08	4.32	11.50	1.28	0.86	1.61	0.22	0.06	0.37	0.48	-0.05	0.99
	Kyoto	7.48	3.07	11.22	1.03	0.65	1.33	0.20	0.09	0.30	0.39	-0.08	0.80
	Mie	7.06	2.95	10.91	1.06	0.58	1.40	0.18	0.08	0.29	0.34	-0.01	0.68
	Miyagi	11.76	5.83	17.10	0.96	0.39	1.38	0.33	0.23	0.44	0.54	-0.07	1.08
	Miyazaki	8.06	4.90	11.17	1.36	0.89	1.68	0.20	0.05	0.33	0.54	-0.04	1.10
	Nagano	12.51	7.02	17.64	1.15	0.64	1.51	0.30	0.19	0.39	0.53	-0.14	1.14
	Nagasaki	8.18	4.88	11.14	1.40	1.02	1.68	0.20	0.07	0.33	0.33	-0.05	0.74
	Nara	7.42	2.49	11.97	0.71	0.20	1.09	0.17	0.10	0.24	0.57	0.17	0.97
	Niigata	10.26	4.25	15.86	0.71	0.23	1.14	0.23	0.15	0.30	0.38	0.08	0.69
	Oita	7.65	3.19	11.83	1.04	0.66	1.35	0.18	0.08	0.27	0.44	0.02	0.85
	Okayama	9.21	2.53	15.05	1.11	0.68	1.45	0.22	0.08	0.35	0.30	-0.28	0.86

	Okinawa	9.07	4.19	13.27	1.36	0.75	1.78	0.20	-0.32	0.69	0.55	-0.49	1.56
	Osaka	6.50	2.96	9.66	0.91	0.56	1.19	0.18	0.06	0.30	0.35	-0.03	0.73
	Saga	7.10	2.33	11.03	1.12	0.68	1.48	0.18	0.05	0.30	0.38	-0.15	0.87
	Saitama	6.81	3.16	10.18	0.95	0.58	1.23	0.22	0.12	0.31	0.47	0.07	0.87
	Shiga	10.01	4.52	14.48	0.87	0.31	1.33	0.27	0.14	0.39	0.50	0.07	0.92
	Shimane	9.19	2.55	15.10	1.16	0.67	1.50	0.21	0.12	0.29	0.31	-0.09	0.75
	Shizuoka	7.40	4.10	10.50	1.10	0.71	1.40	0.15	0.04	0.26	0.60	0.04	1.24
	Tokushima	7.15	3.18	10.86	1.07	0.62	1.41	0.20	0.06	0.33	0.27	-0.17	0.75
	Tochigi	9.70	4.94	14.41	1.14	0.73	1.47	0.31	0.21	0.40	0.65	0.12	1.19
	Tokyo	4.84	0.69	8.92	0.29	-0.12	0.66	0.12	0.04	0.18	0.44	0.05	0.82
	Tottori	8.97	2.14	15.02	0.98	0.51	1.38	0.22	0.11	0.32	0.31	-0.22	0.82
	Toyama	9.86	2.83	15.93	0.85	0.25	1.25	0.20	0.13	0.27	0.31	-0.13	0.71
	Wakayama	7.97	3.80	12.20	1.06	0.60	1.40	0.16	0.07	0.26	0.40	0.03	0.77
	Yamagata	12.25	5.46	17.96	0.81	0.14	1.31	0.28	0.18	0.38	0.46	-0.18	1.13
	Yamaguchi	8.49	3.09	13.60	1.00	0.61	1.34	0.21	0.13	0.30	0.43	-0.04	0.87
	Yamanashi	8.28	3.47	12.33	1.19	0.75	1.57	0.20	0.12	0.27	0.59	0.14	0.99
Korea	Busan	13.11	-4.44	26.24	0.12	-1.86	1.21	0.56	0.06	0.95	0.53	-0.72	1.73
	Daegu	13.75	-2.67	25.44	0.30	-1.36	1.28	0.47	0.04	0.80	0.38	-0.93	1.92
	Daejeon	15.70	-0.89	28.31	0.33	-1.09	1.19	0.45	0.10	0.75	0.21	-1.39	1.73
	Gwangju	14.49	-1.16	27.47	0.22	-1.55	1.18	0.48	0.08	0.79	0.48	-1.04	1.92
	Incheon	15.78	0.44	28.38	0.43	-0.99	1.24	0.47	0.12	0.76	0.07	-1.07	1.27
	Seoul	15.86	-0.16	28.65	0.59	-0.75	1.36	0.49	0.11	0.80	-0.14	-1.84	1.34
	Ulsan	13.41	-5.22	27.52	0.05	-2.13	1.21	0.58	0.08	1.01	0.60	-0.97	2.15
Chinese Taiwan	Kaohsiung	8.38	-2.59	16.81	1.32	0.46	1.83	0.93	0.48	1.29	0.07	-2.47	2.71
	Taipei	11.06	-2.69	21.73	1.35	0.31	1.90	0.83	0.47	1.11	-0.03	-2.70	2.54
	Taichung	10.04	-2.89	20.66	1.56	0.68	2.05	0.80	0.39	1.11	0.01	-3.57	3.33
Vietnam	Ho Chi Minh	29.06	-210.36	56.00	-0.12	-232.03	1.36	0.06	-0.26	0.12	1.63	-13.03	13.90
	Hue	53.18	-795.42	88.42	-0.08	-14.00	0.05	1.04	-28.61	2.69	1.43	-17.33	17.09
The Philippines	Cebu	14.98	-4.96	30.65	0.65	-0.17	1.11	1.07	0.36	1.56	0.99	-3.29	4.62
	Davao	12.99	-4.94	27.87	0.41	-0.46	0.89	1.30	0.44	1.83	1.06	-2.69	4.77
	Manila	13.44	-4.74	28.03	0.75	-0.84	1.47	1.14	0.37	1.60	1.44	-3.57	5.99
	Quezon	17.22	-3.81	33.41	0.78	-1.00	1.49	1.11	0.43	1.61	1.47	-4.41	6.95

Supplementary Table S5. Minimum mortality percentile and temperature by causes of death for each city.

		Minimum Mortality Percentile (MMP) and Temperature (MMT)					
		Non-accidental		Cardiovascular-related		Respiratory-related	
**Country /Region**	**City**	**MMP (%)**	**MMT (℃)**	**MMP (%)**	**MMT (℃)**	**MMP (%)**	**MMT (℃)**
Japan	Aichi	83	25.2	86	26.1	63	19.8
	Akita	92	24.3	92	24.3	91	23.9
	Aomori	92	22.8	93	23.2	92	22.8
	Chiba	92	27	91	26.7	91	26.7
	Ehime	91	27.6	90	27.3	60	19.3
	Fukushima	91	25.3	92	25.7	91	25.3
	Fukui	91	26.8	92	27.1	91	26.8
	Fukuoka	82	25.3	82	25.3	54	18.3
	Gifu	84	25.5	88	26.7	70	21.8
	Gunma	90	26.1	91	26.4	83	23.9
	Hokkaido	92	21.9	92	21.9	84	19.6
	Hiroshima	81	24.6	83	25.3	74	22.7
	Hyogo	74	22.9	90	27.4	63	20.3
	Ibaraki	93	25.8	93	25.8	92	25.5
	Ishikawa	90	26.3	91	26.7	89	25.9
	Iwate	92	23.4	92	23.4	92	23.4
	Kagawa	84	25.8	90	27.4	67	21.2
	Kanagawa	91	26.7	91	26.7	91	26.7
	Kagoshima	73	24.2	82	26.8	54	19.9
	Kochi	72	22.8	91	27.3	66	21.4
	Kumamoto	71	22.9	90	27.7	63	21
	Kyoto	86	26.4	90	27.6	66	20.8
	Mie	82	24.8	90	26.8	65	20.2
	Miyagi	94	24.9	94	24.9	92	24.2
	Miyazaki	74	23.5	91	27.5	58	19.9
	Nagano	92	25.2	92	25.2	91	24.9
	Nagasaki	82	25.5	86	26.6	60	20
	Nara	92	26.9	92	26.9	91	26.6
	Niigata	90	25.4	91	25.8	86	24.1
	Oita	92	27.4	91	27.1	68	21.1
	Okayama	91	27.9	90	27.6	75	23.2
	Okinawa	99	30	85	28.3	50	23.3
	Osaka	80	25.3	83	26.2	64	21
	Saga	81	25.2	91	27.7	65	21
	Saitama	88	25.8	91	26.7	64	19.1

	Shiga	91	26.8	92	27	91	26.8
	Shimane	90	26	90	26	77	22.2
	Shizuoka	92	27	93	27.2	61	19.6
	Tokushima	82	25.2	86	26.2	62	20.1
	Tochigi	92	25.9	92	25.9	92	25.9
	Tokyo	88	26.6	87	26.2	91	27.4
	Tottori	90	26.4	91	26.7	78	22.6
	Toyama	86	24.4	91	26.1	81	22.9
	Wakayama	79	24.7	90	27.6	66	21.2
	Yamagata	91	24.6	92	24.9	87	23.2
	Yamaguchi	86	25.6	91	26.9	75	22.55
	Yamanashi	91	26.4	91	26.4	66	19.7
Korea	Busan	94	26.6	93	26.2	84	23.3
	Daegu	91	26.9	91	26.9	81	23.8
	Daejeon	91	25.5	92	25.8	80	23.0
	Gwangju	92	26.5	92	26.5	81	23.7
	Incheon	93	25.4	93	25.4	80	22.3
	Seoul	84	24.0	85	24.2	79	22.8
	Ulsan	93	26.5	92	26.2	83	23.4
Chinese Taiwan	Kaohsiung	45	25.7	76	28.4	69	27.9
	Taipei	57	25.0	76	27.8	76	27.8
	Taichung	51	25.0	77	27.8	75	27.6
Vietnam	Ho Chi Minh	83	29.8	82	29.7	99	31.6
	Hue	79	29.3	8	18.7	1	15.8
The Philippines	Cebu	79	29.7	76	29.6	6	26.3
	Davao	80	29.0	77	28.9	5	25.8
	Manila	79	29.2	1	24.9	6	26.5
	Quezon	79	28.6	72	28.2	6	25.0

Supplementary Table S6. Random-effect meta-regression model at second stage with multivariate Cochran Q test for heterogeneity (p-value), and I^2 statistics (%) in various models (for first step).

Deaths	Relationship	Model	Predictor	Q test	I^2
**Non-accidental**	Temperature-mortality	Intercept-only	-	<0.0001	57.3%
		Single predictor	Average temperature	<0.0001	52.3%
			Temperature range	<0.0001	55.2%
			Country	<0.0001	53.0%
		Full model	Average temperature<br>Temperature range<br>Country	<0.0001	40.8%
	DTR-mortality	Intercept-only	-	1.0000	1.0%
		Single predictor	Average temperature	1.0000	1.0%
			Temperature range	1.0000	1.0%
			Country	1.0000	1.0%
		Full model	Average temperature	1.0000	1.0%
			Temperature range	1.0000	1.0%
			Country	1.0000	1.0%
**Cardiovascular-related**	Temperature-mortality	Intercept-only	-	<0.0001	42.5%
		Single predictor	Average temperature	<0.0001	37.0%
			Temperature range	<0.0001	40.7%
			Country	<0.0001	38.9%
		Full model	Average temperature<br>Temperature range<br>Country	<0.0001	26.8%
	DTR-mortality	Intercept-only	-	1.0000	1.0%
		Single predictor	Average temperature	1.0000	1.0%
			Temperature range	1.0000	1.0%
			Country	1.0000	1.0%
		Full model	Average temperature	1.0000	1.0%
			Temperature range	1.0000	1.0%
			Country	1.0000	1.0%
**Respiratory-related**	Temperature-mortality	Intercept-only	-	<0.0001	28.2%
		Single predictor	Average temperature	0.0005	22.5%
			Temperature range	<0.0001	26.1%
			Country	0.0002	24.4%
		Full model	Average temperature<br>Temperature range<br>Country	0.0425	13.1%
	DTR-mortality	Intercept-only	-	1.0000	1.0%
		Single predictor	Average temperature	1.0000	1.0%
			Temperature range	1.0000	1.0%
			Country	1.0000	1.0%
		Full model	Average temperature	1.0000	1.0%
			Temperature range	1.0000	1.0%
			Country	1.0000	1.0%

Supplementary Table S7. Study periods and data sources for each city

Country /Region	City	Study period	Mortality	Weather variable
**Japan**	47 prefectures	1972–2012	Ministry of Health & Welfare of Japan	Japan Meteorological Agency
**South Korea**	7 cities	1992–2010	Korea National Statistics Office	Korea Meteorological Office
**Chinese Taiwan**	3 cities	1994–2007	National Death Registry of Taiwan	Taiwan Environmental Protection Administration
**Vietnam**	2 cities	2009-2013	Provincial Health Departments	US National Oceanic and Atmospheric Administration's National Climate Data Center
**The Philippines**	4 cities	2006-2010	Philippine Statistics Authority - National Statistics Office	US National Oceanic and Atmospheric Administration's National Climate Data Center

Supplementary Figure Legends

Supplementary Figure S1. Total (non-accidental) mortality. Cumulative exposure-response relations in 63 east-Asia locations: Exposure-response associations with related temperature. Thick dashed lines are the 2.5th and 97.5th percentiles as cut offs. Light dashed lines are minimum mortality temperatures**.** Using city-specific distributed lag non-linear model.

Supplementary Figure S2. Lag-response associations based on 99% diurnal temperature range(°C) in 63 east-Asia locations for total (non-accidental) mortality: Lag-response associations from predicted values of multivariate meta-regression (with 95% confidence intervals, vertical bar) in three countries. RR, relative risk.

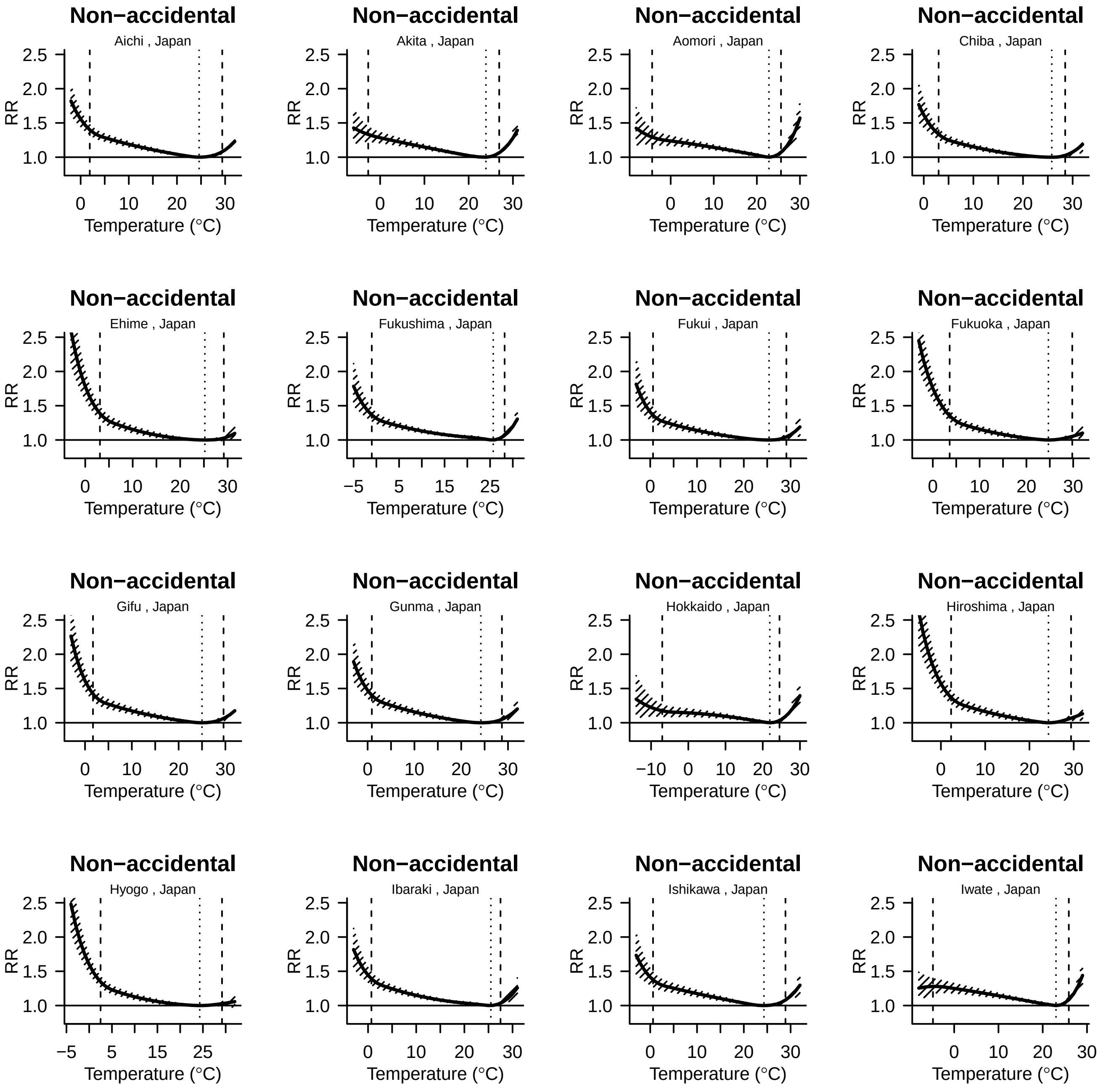
Non−accidental
Aichi , Japan
Akita , Japan
Aomori , Japan
Chiba , Japan
Ehime , Japan
Fukushima , Japan
Fukui , Japan
Fukuoka , Japan
Gifu , Japan
Gunma , Japan
Hokkaido , Japan
Hiroshima , Japan
Hyogo , Japan
Ibaraki , Japan
Ishikawa , Japan
Iwate , Japan
RR
Temperature (°C)

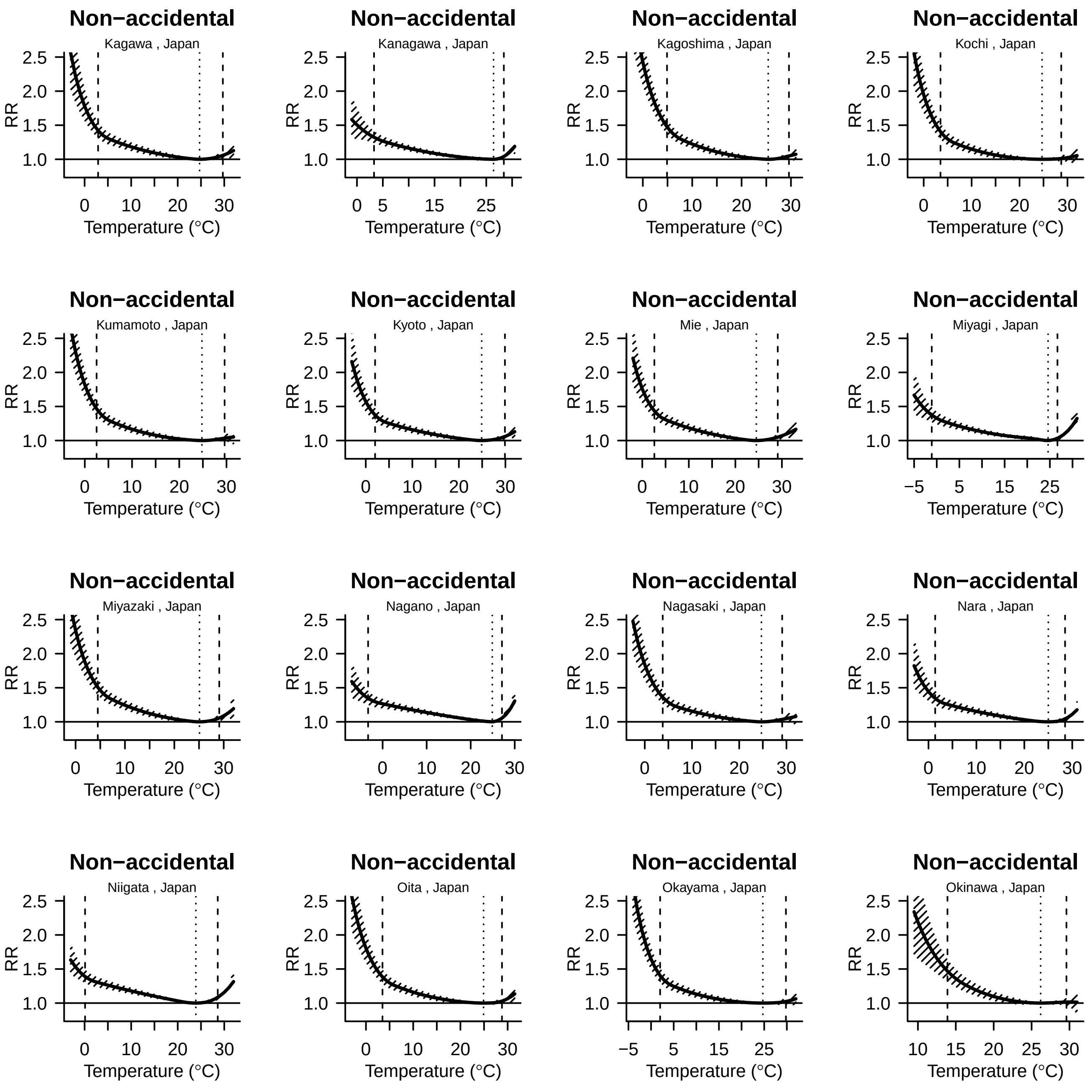
Non−accidental
Kagawa , Japan
Non−accidental
Kanagawa , Japan
Non−accidental
Kagoshima , Japan
Non−accidental
Kochi , Japan
Non−accidental
Kumamoto , Japan
Non−accidental
Kyoto , Japan
Non−accidental
Mie , Japan
Non−accidental
Miyagi , Japan
Non−accidental
Miyazaki , Japan
Non−accidental
Nagano , Japan
Non−accidental
Nagasaki , Japan
Non−accidental
Nara , Japan
Non−accidental
Niigata , Japan
Non−accidental
Oita , Japan
Non−accidental
Okayama , Japan
Non−accidental
Okinawa , Japan
RR
Temperature (°C)

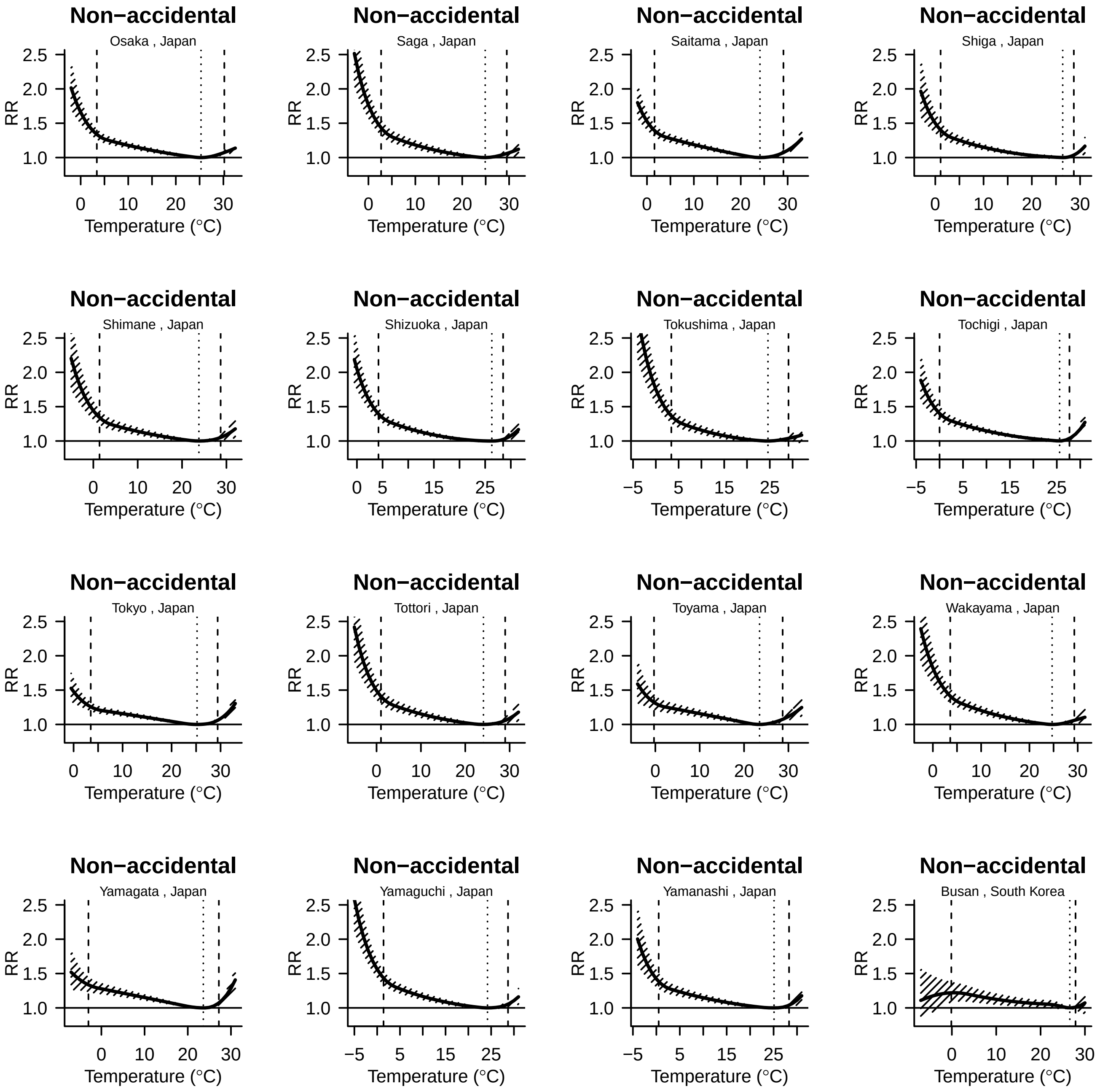
Non−accidental
Osaka , Japan
Non−accidental
Saga , Japan
Non−accidental
Saitama , Japan
Non−accidental
Shiga , Japan
Non−accidental
Shimane , Japan
Non−accidental
Shizuoka , Japan
Non−accidental
Tokushima , Japan
Non−accidental
Tochigi , Japan
Non−accidental
Tokyo , Japan
Non−accidental
Tottori , Japan
Non−accidental
Toyama , Japan
Non−accidental
Wakayama , Japan
Non−accidental
Yamagata , Japan
Non−accidental
Yamaguchi , Japan
Non−accidental
Yamanashi , Japan
Non−accidental
Busan , South Korea
RR
Temperature (°C)

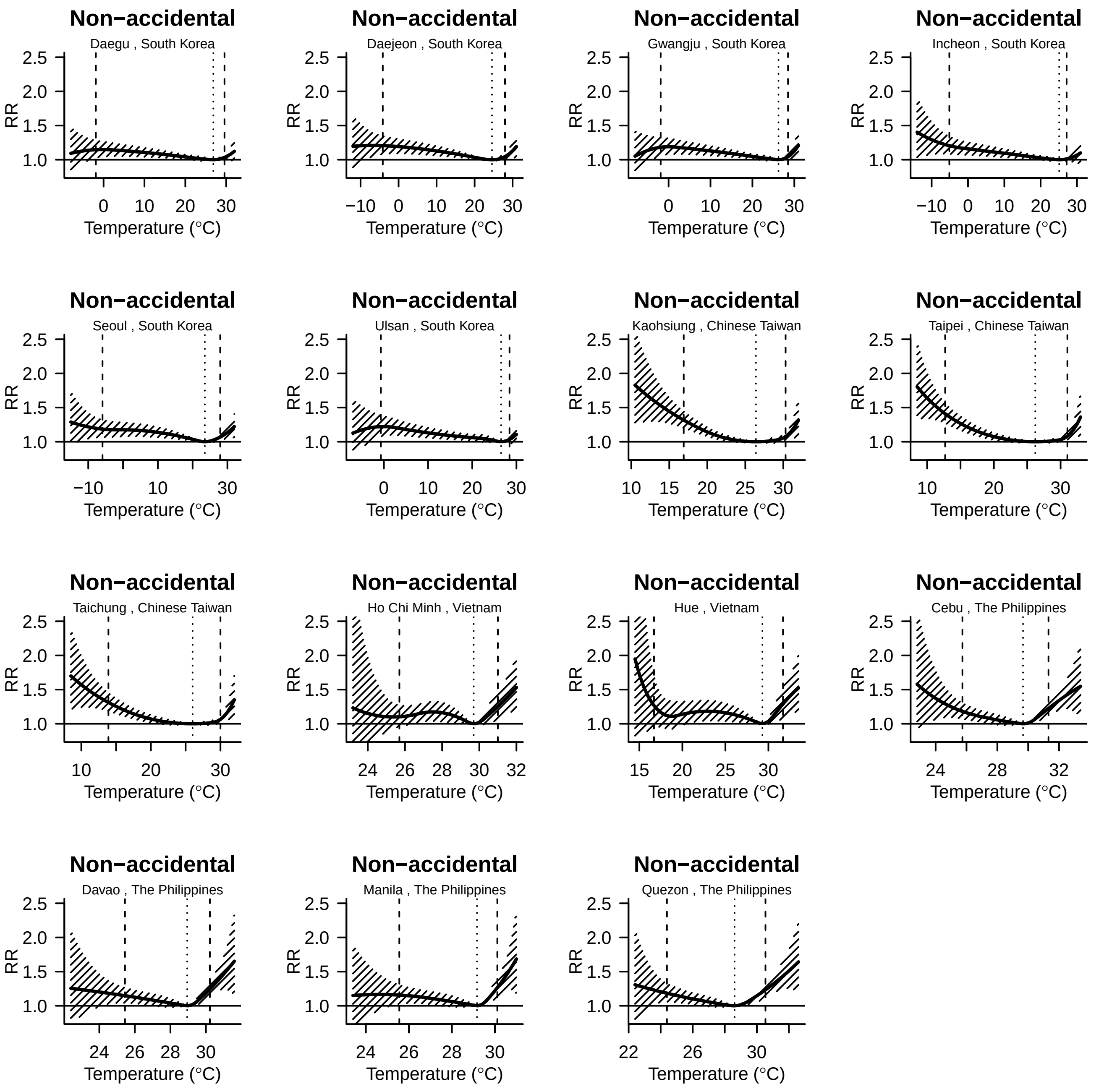
Non−accidental
Daegu , South Korea
Non−accidental
Daejeon , South Korea
Non−accidental
Gwangju , South Korea
Non−accidental
Incheon , South Korea
Non−accidental
Seoul , South Korea
Non−accidental
Ulsan , South Korea
Non−accidental
Kaohsiung , Chinese Taiwan
Non−accidental
Taipei , Chinese Taiwan
Non−accidental
Taichung , Chinese Taiwan
Non−accidental
Ho Chi Minh , Vietnam
Non−accidental
Hue , Vietnam
Non−accidental
Cebu , The Philippines
Non−accidental
Davao , The Philippines
Non−accidental
Manila , The Philippines
Non−accidental
Quezon , The Philippines
RR
Temperature (°C)

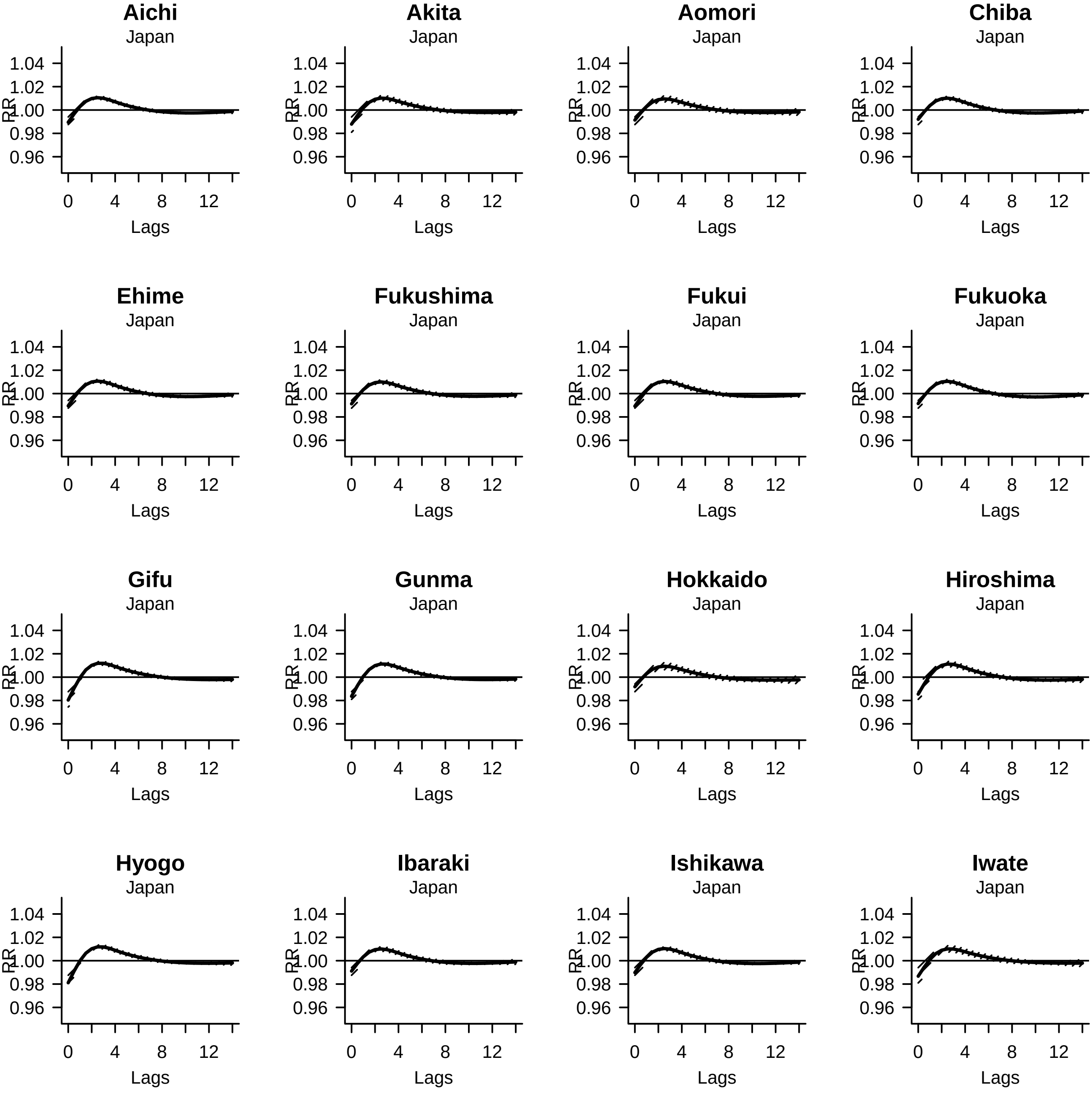
Aichi
Japan
Akita
Japan
Aomori
Japan
Chiba
Japan
Ehime
Japan
Fukushima
Japan
Fukui
Japan
Fukuoka
Japan
Gifu
Japan
Gunma
Japan
Hokkaido
Japan
Hiroshima
Japan
Hyogo
Japan
Ibaraki
Japan
Ishikawa
Japan
Iwate
Japan
RR
1.04
1.02
1.00
0.98
0.96
Lags
0
4
8
12

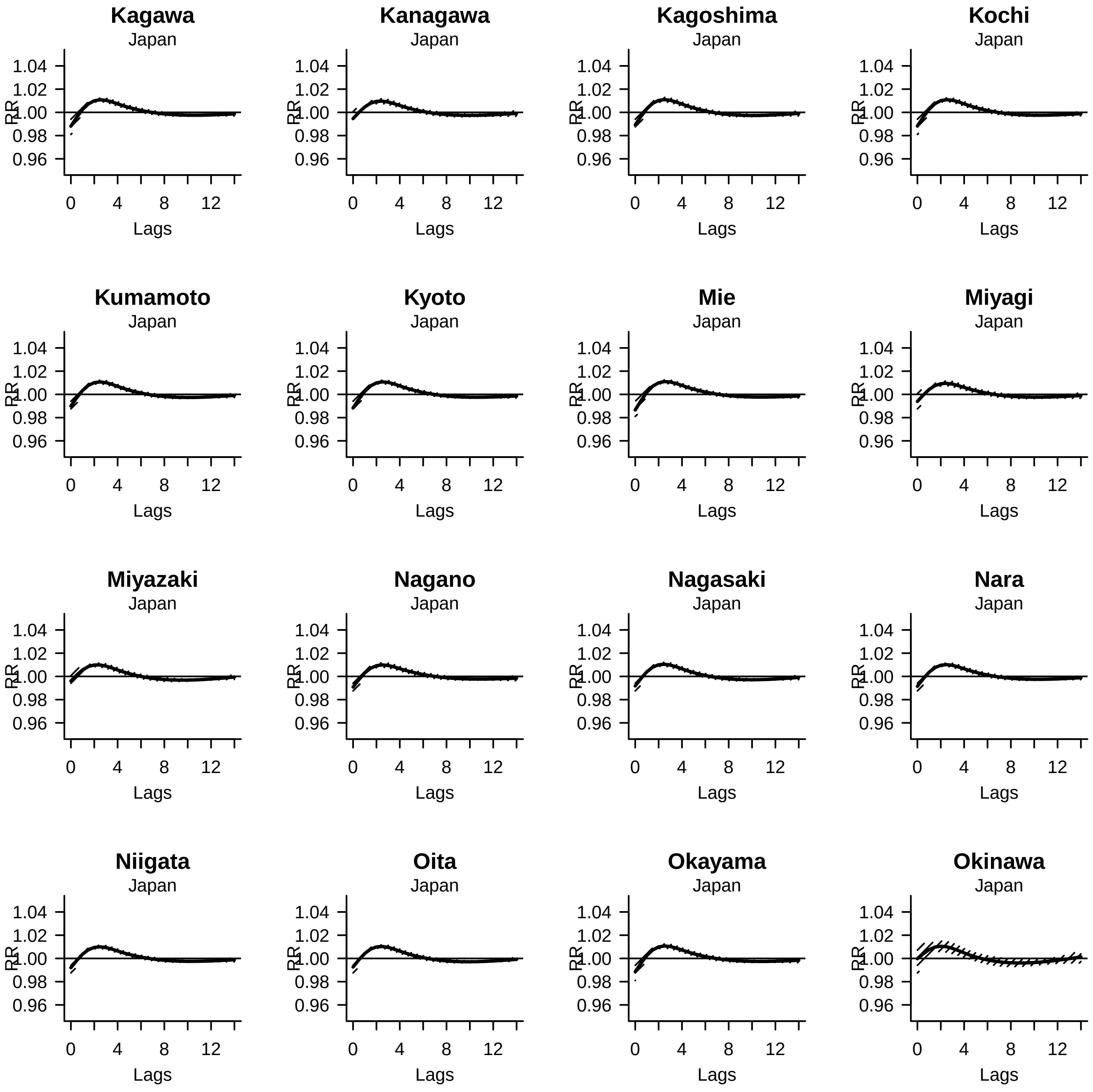

Kagawa
Japan
Kanagawa
Japan
Kagoshima
Japan
Kochi
Japan
Kumamoto
Japan
Kyoto
Japan
Mie
Japan
Miyagi
Japan
Miyazaki
Japan
Nagano
Japan
Nagasaki
Japan
Nara
Japan
Niigata
Japan
Oita
Japan
Okayama
Japan
Okinawa
Japan
RR
1.04
1.02
1.00
0.98
0.96
0
4
8
12
Lags

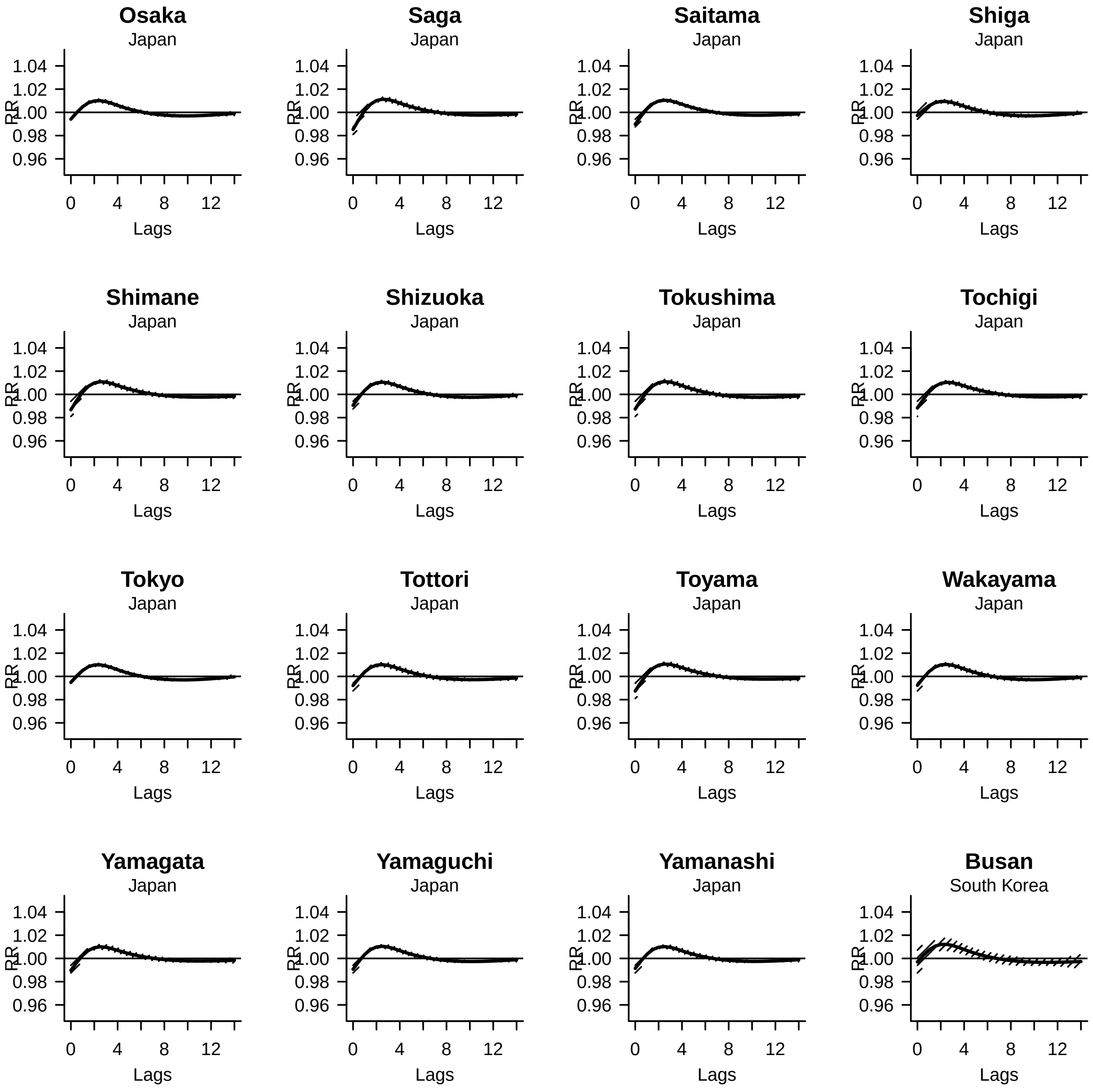
Osaka
Japan
Saga
Japan
Saitama
Japan
Shiga
Japan
Shimane
Japan
Shizuoka
Japan
Tokushima
Japan
Tochigi
Japan
Tokyo
Japan
Tottori
Japan
Toyama
Japan
Wakayama
Japan
Yamagata
Japan
Yamaguchi
Japan
Yamanashi
Japan
Busan
South Korea
RR
1.04
1.02
1.00
0.98
0.96
Lags
0
4
8
12

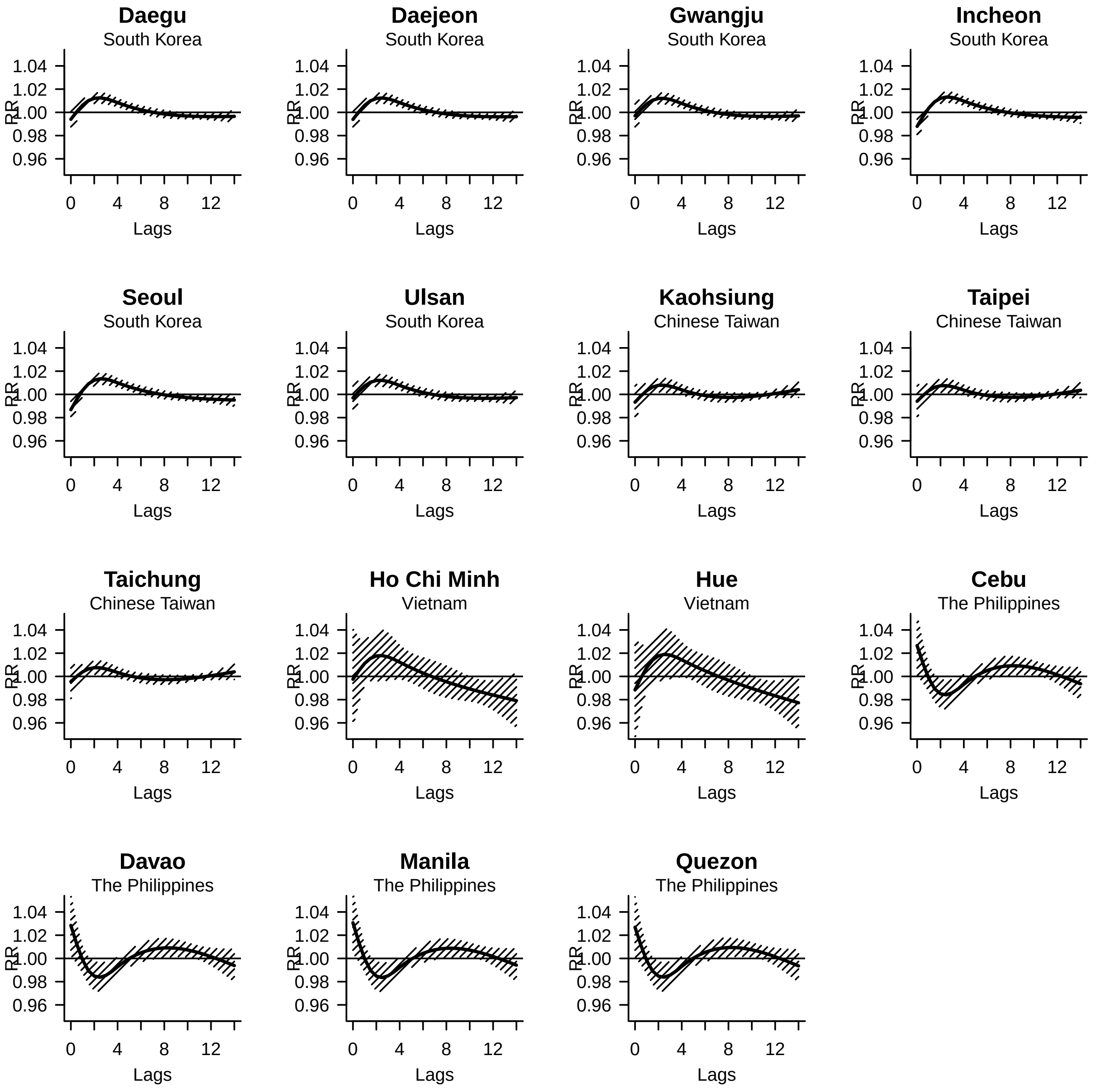
Daegu
South Korea
Daejeon
South Korea
Gwangju
South Korea
Incheon
South Korea
Seoul
South Korea
Ulsan
South Korea
Kaohsiung
Chinese Taiwan
Taipei
Chinese Taiwan
Taichung
Chinese Taiwan
Ho Chi Minh
Vietnam
Hue
Vietnam
Cebu
The Philippines
Davao
The Philippines
Manila
The Philippines
Quezon
The Philippines
RR
1.04
1.02
1.00
0.98
0.96
Lags
0
4
8
12
